# Facilitated receptor-recognition and enhanced bioactivity of bone morphogenetic protein-2 on magnesium-substituted hydroxyapatite surface

**DOI:** 10.1038/srep24323

**Published:** 2016-04-14

**Authors:** Baolin Huang, Yuan Yuan, Tong Li, Sai Ding, Wenjing Zhang, Yuantong Gu, Changsheng Liu

**Affiliations:** 1 State Key Laboratory of Bioreactor Engineering, East China University of Science and Technology, Shanghai 200237, PR China; 2 Key Laboratory for Ultrafine Materials of Ministry of Education, East China University of Science and Technology, Shanghai 200237, PR China; 3 School of Chemistry, Physics and Mechanical Engineering, Queensland University of Technology, 2 George St, Brisbane, QLD 4001, Australia; 4 Engineering Research Center for Biomedical Materials of Ministry of Education, East China University of Science and Technology, Shanghai 200237, PR China

## Abstract

Biomaterial surface functionalized with bone morphogenetic protein-2 (BMP-2) is a promising approach to fabricating successful orthopedic implants/scaffolds. However, the bioactivity of BMP-2 on material surfaces is still far from satisfactory and the mechanism of related protein-surface interaction remains elusive. Based on the most widely used bone-implants/scaffolds material, hydroxyapatite (HAP), we developed a matrix of magnesium-substituted HAP (Mg-HAP, 2.2 at% substitution) to address these issues. Further, we investigated the adsorption dynamics, BMPRs-recruitment, and bioactivity of recombinant human BMP-2 (rhBMP-2) on the HAP and Mg-HAP surfaces. To elucidate the mechanism, molecular dynamic simulations were performed to calculate the preferred orientations, conformation changes, and cysteine-knot stabilities of adsorbed BMP-2 molecules. The results showed that rhBMP-2 on the Mg-HAP surface exhibited greater bioactivity, evidenced by more facilitated BMPRs-recognition and higher ALP activity than on the HAP surface. Moreover, molecular simulations indicated that BMP-2 favoured distinct side-on orientations on the HAP and Mg-HAP surfaces. Intriguingly, BMP-2 on the Mg-HAP surface largely preserved the active protein structure evidenced by more stable cysteine-knots than on the HAP surface. These findings explicitly clarify the mechanism of BMP-2-HAP/Mg-HAP interactions and highlight the promising application of Mg-HAP/BMP-2 matrixes in bone regeneration implants/scaffolds.

The healing of spinal fusions, bone defects, and fractures of bone are still great challenges in recent years^1^,^2^. To address these issues, loading of growth factors into implantable scaffolds is a well-established and promising avenue to reconstitute normal fracture healing and hereby enhance union^3^,^4^,^5^. Among the growth factors, bone morphogenetic protein-2 (BMP-2) from the transforming growth factor-β (TGF-β) superfamily has been identified as a potent osteogenic growth factor to induce bone formation^6^,^7^,^8^ and was approved by US Food and Drug Administration for clinical applications in 2002^1^,^6^. Although the use of BMP-2 enhances fracture healing, the bioactivity of BMP-2 loaded in delivery systems is still need to be modulated to induce robust bone regeneration due to a short half-life and an improper immobilization^1^,^7^,^8^. Moreover, many previous investigations found that the hydrogen/ionic/hydrophobic interactions often lead to changes in secondary/tertiary structure and denaturation of BMP-2 both *in vitro* and *in vivo*^7^,^9^,^10^. This directly undermines its therapeutic efficacy and thus results in a high-dosage usage accompanied with high therapy costs and undesirable side effects^2^,^3^,^8^. Therefore, there is an urgent need to understand and tailor the adsorption and bioactivity of BMP-2 upon orthopedic implants/scaffolds.

Recently, the interactions between protein and biomaterial surface have attracted great attention due to its significance in many research fields^11^,^12^,^13^,^14^,^15^. Plenty of studies have indicated that surface parameters (e.g. charge^16^, chemical composition^17^, topography^18^, and hydrophilicity^19^) on the nanoscale can largely affect protein adsorption, conformation, and biological activity on material surfaces. Specifically, the surface-mediated changes of adsorption and bioactivity of recombinant human BMP-2 (rhBMP-2) have been demonstrated by some recent studies^20^,^21^,^22^,^23^,^24^. For example, negatively charged graphene oxide sheets could load large doses of rhBMP-2 and preserve the structure and bioactivity of the protein^10^. Also, TiO_2_ nanotube surfaces immobilized with rhBMP-2 have shown good biological performance evidenced by great differentiation and low apoptosis of bone stromal stem cells (BMSCs) cultured on these surfaces^22^. These studies highlight the possibility to mediate the bioactivity of adsorbed rhBMP-2 molecules by tailoring its binding mode. However, to date, the effect of the binding mode of rhBMP-2 to bone morphogenetic protein receptors (BMPRs)-recognition is still poorly understood, and the mechanism involved for the ensuring bioactivity of rhBMP-2 is not clear.

In recent years, a rash of biomaterials (e.g. collagen^1^,^5^, graphene^10^, heparin^20^,^25^, apatite^21^, and titanium^22^,^26^) have been extensively exploited for loading rhBMP-2 cargos in bone regeneration. Among these vehicles, hydroxyapatite [HAP, Ca_10_(PO_4_)_6_(OH)_2_] is the most extensively applied biocompatible ceramic materials as its chemical composition is similar to the mineral of hard tissues^15^,^21^. Moreover, due to favourable interactions with native bone tissue, HAP is often considered as the “golden standard” in orthopaedics^27^. To enhance the biological activity, synthetic HAP was usually doped with small amounts of additives, such as Mg^2+^, Zn^2+^, Sr^2+^, CO_3_^2−^, SiO_4_^4−^, and F^−^27,28,29. These small species not only changed the morphology, stability, and solubility of HAP, but also affected the biological response of adsorbed proteins^27^,^28^. One of the most important elements is magnesium (Mg), which is the fourth most abundant cation in human body^30^,^31^. It was found that a low concentration of Mg^2+^ played a crucial role in bone metabolism, and its depletion caused bone fragility and bone loss^32^. In addition, one recent study has found that the substitution of Mg^2+^ to Ca^2+^ (even low content) in calcium phosphate cement modulated adsorption of fibronectin and thus ameliorated the cellular response of BMSCs^33^. Therefore, it is interesting to investigate the potential positive effects of adsorption and bioactivity of rhBMP-2 on Mg-substituted HAP (Mg-HAP) surface compared to the HAP surface and the involved mechanism.

Currently, molecular dynamics (MD) simulations provide many new insights into the understanding of protein adsorption on nanoscale surfaces at atomic level^34^,^35^. Moreover, steered molecular dynamics (SMD) simulation is developed to accelerate the processes of adsorption and/or desorption^36^,^37^,^38^. These two simulations have been combined to investigate the adsorption dynamics of proteins on many surfaces. For instance, a series of MD and SMD simulations showed a shield effect in the adsorption processes of leucine-rich amelogenin protein onto silicon-doped HAP surfaces^37^. In another recent study using MD and SMD, it has found that the interfacial mechanical behavior is governed by the electrostatic attraction between an osteopontin and a HAP surface^38^. In addition, a series of studies investigated the adsorption of bone morphogenetic proteins (BMPs) on the HAP surface^36^,^39^. It was proven that proteins could show different adsorption mechanisms on the HAP surface, such as the adsorption formed with electrostatic interaction and water-bridged H-bonds^36^,^39^. These works clearly explained the conformational changes of the protein during the adsorption/desorption processes, but they lack *in vitro/vivo* validation of the bioactivity of the adsorbed proteins. Despite all these MD and SMD simulations for BMP-2 adsorption, up to date, relatively little knowledge has been obtained about the BMPRs-recruitment and bioactivity of BMP-2 upon the HAP and Mg-HAP model surfaces.

Therefore, we investigated the adsorption behaviour, recognition of BMPRs, and bioactivity of rhBMP-2 on the HAP and Mg-HAP surfaces by quartz crystal microbalance with dissipation (QCM-D) experiments, cell experiments, and MD/SMD simulations. To prepare the respective surfaces, HAP and Mg-HAP nanocrystals were fabricated by a microwave method and deposited with an electrophoretic deposition (EPD) method. The adsorption dynamics and recruitments of BMPRs were examined with a QCM-D technique. The bioactivity of rhBMP-2 was measured by an alkaline phosphatase (ALP) activity study with C2C12 cells and BMSCs. In addition, combined MD and SMD simulations were carried out to simulate 6 classical orientations of BMP-2 adsorbed on the HAP and Mg-HAP model surfaces. The detailed mechanism was also elucidated by the experimental results and numerical simulations.

## Results

### Characterization of the HAP and Mg-HAP nanoparticles

**Figure 1:**
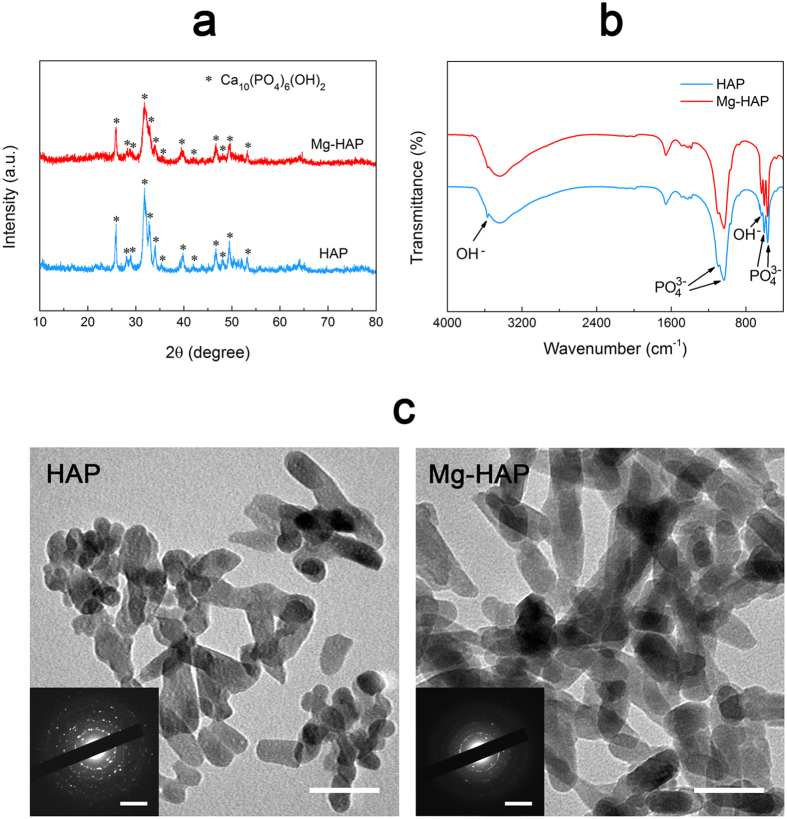
XRD patterns (**a**) FTIR spectra (**b**) and TEM and SAED images (**c**) of the HAP and Mg-HAP nanoparticles. Scale bars are 50 nm for TEM images, and 30 nm for SAED patterns.

Phase compositions of the HAP and Mg-HAP nanoparticles were characterized by X-ray diffraction (XRD). As shown in Fig. 1a, the peaks of stoichiometric HAP are indexed according to a standard pattern (JCPDS 09-0423). It can be observed that the HAP nanoparticles exhibit sharp diffraction peaks, and the intensity of peaks of the Mg-HAP nanoparticles is lower. As shown in Fig. 1b, all Fourier transform infrared (FTIR) spectra of the HAP and Mg-HAP nanocrystals illustrate OH- bands at 656 and 3569 cm^−1^ and PO_4_^3−^ bands at 565, 603, 1032, and1089 cm^−1^. Transmission electron microscope (TEM) images and selected-area diffraction patterns (SAED) revealed that the HAP and Mg-HAP nanocrystals were rod-like and typical polycrystalline (Fig. 1c). Moreover, zeta potentials of the HAP and Mg-HAP nanocrystals were −1.4 ± 0.3 mV and −0.9 ± 0.4 mV (n = 5), respectively. Energy dispersive X-ray spectra (EDS) patterns of the HAP and Mg-HAP nanoparticles showed strong peaks of Ca, P, and O (Fig. S1, Supporting Information). Notably, a peak of Mg was found in the EDS pattern of Mg-HAP. In addition, the ratio of Mg/Ca was confirmed as 2.1 at% for the Mg-HAP nanocrystals.

### Characterization of the HAP and Mg-HAP surfaces

**Figure 2:**
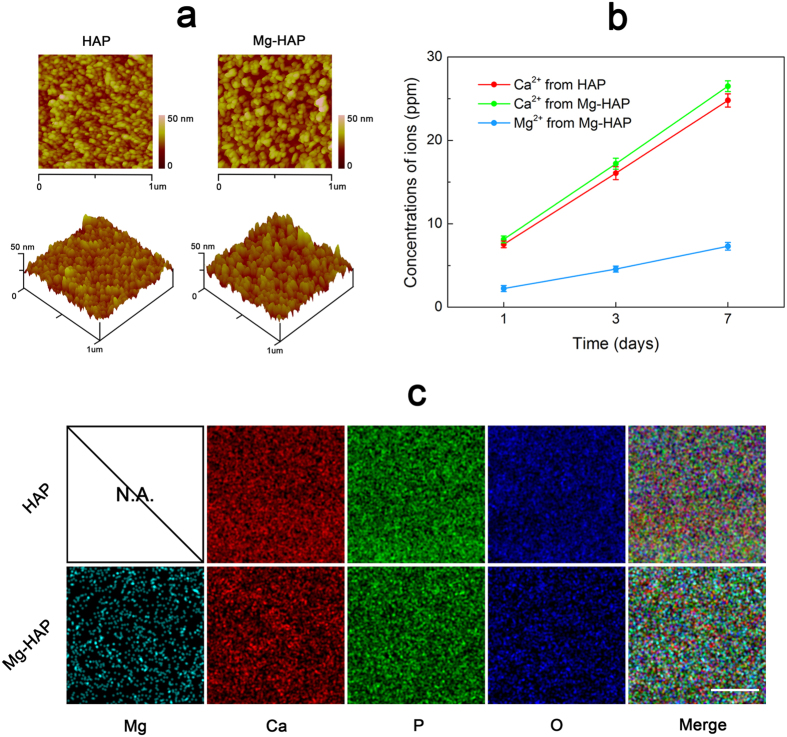
(**a**) AFM images of the HAP and Mg-HAP surfaces. (**b**) Mg^2+^ and Ca^2+^ ions released from the HAP and Mg-HAP surfaces. (**c**) Element distributions on the HAP and Mg-HAP surfaces. N.A. represents not available. Scale bar is 5 um.

Surface topographies of the HAP and Mg-HAP surfaces were evaluated by atomic force microscopy (AFM, Fig. 2a). It can be found that the HAP and Mg-HAP surfaces consist of very delicate nanostructures. Thickness and Ca/P ratio of coatings, root-mean-square roughness (RMS), surface potential, and water contact angle of the HAP and Mg-HAP surfaces are reported in Table 1. Surface morphology observed by scanning electron microscopy (SEM, Fig. S2a, Supporting Information) showed that the HAP and Mg-HAP nanoparticles were uniformly deposited on the respective surfaces. EDS patterns (Fig. S2b,c, Supporting Information) of the deposited coatings revealed similar element contents as compared to those of respective nanoparticles. In particular, the ratio of Mg/Ca was demonstrated as 2.2 at% for the Mg-HAP coatings. Importantly, this low amount of substitution of Mg to Ca was suggested as an optimum concentration for exploring protein adsorption according to literature^30^,^31^. The concentration curves of Mg^2+^ and Ca^2+^ ions released from the HAP and Mg-HAP surfaces are shown in Fig. 2b. It can be clearly found that Ca^2+^ ions released from the HAP and Mg-HAP surfaces are not significantly different (p > 0.05) and gradually increased with time. The release of Mg^2+^ions was only detected in the Mg-HAP surface and slowly increased with time. It is worthwhile to mention that the discrepancy of Ca^2+^ release between the HAP and Mg-HAP samples is inconspicuous, which was due to the EPD fabrication process mediated the ion-release rate of the HAP and Mg-HAP coatings as compared to their nanoparticle state. Moreover, the element distributions of the HAP and Mg-HAP surfaces are presented in Fig. 2c. It suggested that Mg, Ca, P, and O were uniformly located on the respective surfaces. Notably, Mg was sparsely distributed on the Mg-HAP surface.

### Adsorption dynamic of rhBMP-2 and recognition of BMPRs

**Figure 3:**
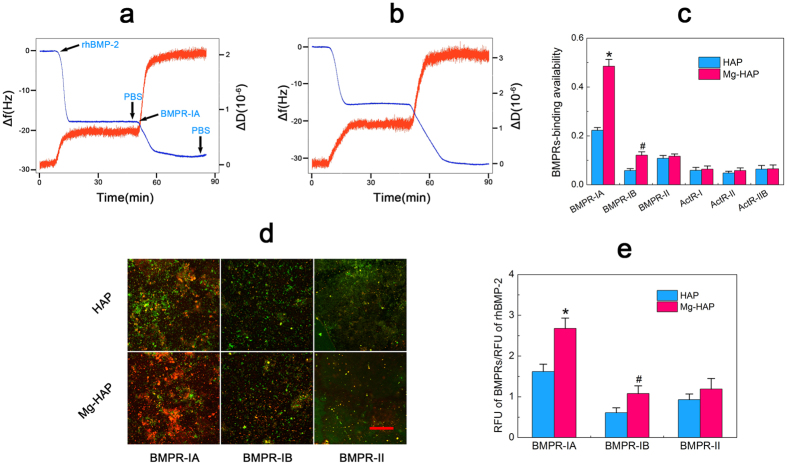
Typical changes in Δf (blue line) and ΔD (red line) recorded against time during the adsorption of rhBMP-2 and subsequent binding of BMPR-IA on (**a**) the HAP surface and (**b**) the Mg-HAP surface. (**c**) Calculated BMPRs-binding availability by adsorbed rhBMP-2 on the HAP and Mg-HAP surfaces. Values are shown as mean ± standard error of the mean from 5 data points (n = 5). ^*^p < 0.05, ^#^*p* < 0.05, compared with the HAP surface. (**d**) CLSM observation of the fluorescent staining of adsorbed rhBMP-2 (green) and subsequent recruited BMPRs (red) on the HAP and Mg-HAP surfaces. Scale bar is 50 um. (**e**) The RFU (relative fluorescence units) of BMPRs per RFU of rhBMP-2. Values are shown as mean ± standard error of the mean from 50 data points (n = 50). ^*^p < 0.05, ^#^*p* < 0.05, compared with the HAP surface.

We measured and compared the adsorption of rhBMP-2 and subsequent recognition of BMPRs on the HAP and Mg-HAP surfaces. Typical QCM-D shifts in frequency and dissipation for the adsorption of rhBMP-2 and subsequent binding of BMPR-IA are shown in Fig. 3a,b. The adsorption amounts of rhBMP-2 and BMPRs were calculated with the Sauerbrey equation^11^ (Table 2). It was found that the Mg-HAP surface showed a slightly lower mass-uptake of rhBMP-2 than the HAP surface. However, the adsorbed rhBMP-2 on the Mg-HAP surface induced an increased recruitment of BMPR-IA/B. For example, a nearly 1.9-fold mass-uptake of BMPR-IA and 1.8-fold mass-uptake of BMPR-IB was found. To further compare the capacity of the adsorbed rhBMP-2 for recruiting BMPRs, the “BMPRs-binding availability” was calculated (Fig. 3c). As expected, a nearly 2.2-time availability of BMPR-IA and 2.1-time availability of BMPR-IB was achieved for adsorbed rhBMP-2 on the Mg-HAP surface. There was no significant difference (p > 0.05) for the binding availability of BMPR-II by adsorbed rhBMP-2 on both surfaces. The binding amounts of ActR-I, ActR-II, and ActR-IIB by adsorbed rhBMP-2 were very low (Table 2), and their binding availabilities were not significantly different (p > 0.05) between the HAP/rhBMP-2 and Mg-HAP/rhBMP-2 surfaces (Fig. 3c). Therefore, we focused on BMPR-IA, BMPR-IB, and BMPR-II of BMPRs in recognition with rhBMP-2. Additionally, we reported the binding kinetics and maximum binding capabilities of rhBMP-2 on the HAP and Mg-HAP surfaces (Fig. S3, Supporting Information), which suggested that the adsorption of rhBMP-2 fitted well with the well-known classical Langmuir adsorption theory^9^,^11^.

To further identify the recognition of BMPRs by adsorbed rhBMP-2, we measured the immunofluorescence staining of rhBMP-2 and BMPRs (Fig. 3d). There is no significant difference (p > 0.05) between the binding availability of TRITC-BMPRs/FITC-rhBMP-2 and that of BMPRs/rhBMP-2 (Fig. S4, Supporting Information). Compared with the HAP surface, large recognized BMPR-IA clusters were achieved on the Mg-HAP surface. The clusters of BMPR-IB on the HAP surface were small (<5 um) and uniformly dispersed, while that on the Mg-HAP surface exhibited large aggregations (10–15 um). The clusters of BMPR-II on both surfaces were comparable (5–10 um). In addition, the relative fluorescence units (RFU) of BMPRs per RFU of rhBMP-2 were calculated (Fig. 3e). It showed a similar trend as compared to the results of “BMPRs-binding availability”. For instance, an almost 1.7-time recruitment of BMPR-IA per rhBMP-2, 1.8-time recruitment BMPR-IB per rhBMP-2, and comparable recruitment of BMPR-II per rhBMP-2 were achieved on the Mg-HAP surface as compared to those on the HAP surface.

### Adsorption stability and bioactivity of rhBMP-2

**Figure 4:**
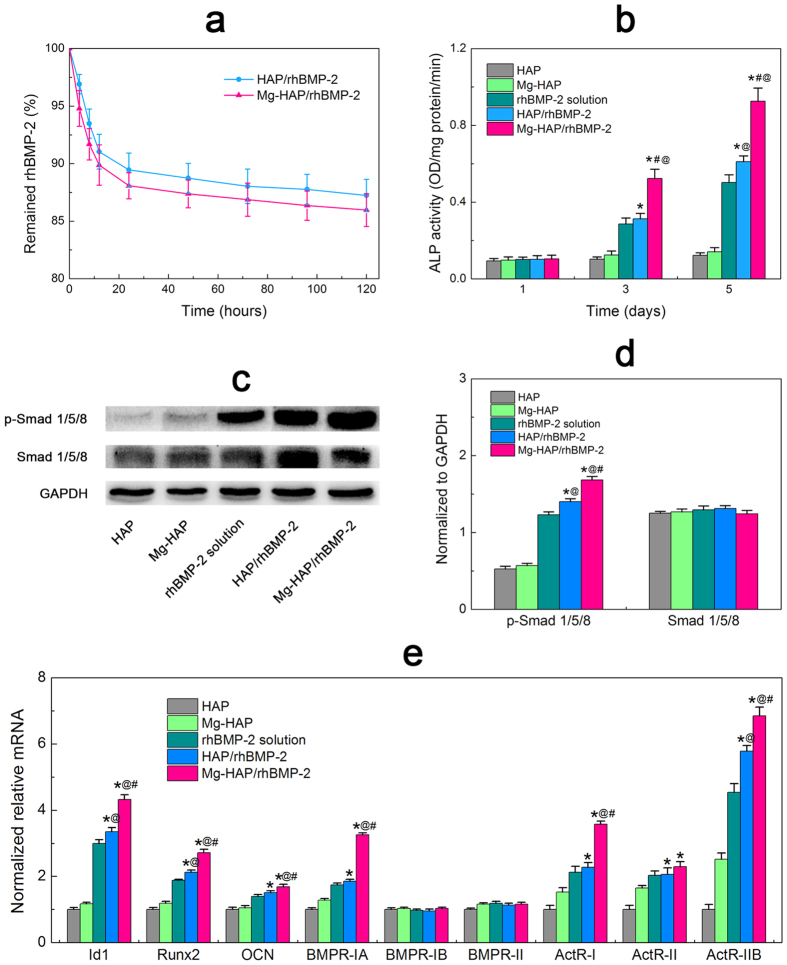
(**a**) Remained rhBMP-2 on the HAP and Mg-HAP surfaces. Values are shown as mean ± standard error of the mean from 6 data points (n = 6). (**b**) ALP activity assay. The HAP and Mg-HAP surfaces without rhBMP-2 were acted as negative controls. The rhBMP-2 solution representing culture medium containing fresh rhBMP-2 at the similar content to that calculated from the QCM-D study was used as a positive control. (**c**) Western blot of p-Smad1/5/8, Smad1/5/8, and GAPDH for C2C12 cells cultured on respective surfaces. (**d**) Relative expressions of p-Smad1/5/8 and Smad1/5/8 (data are normalized to the total GAPDH content). Values are shown as mean ± standard error of the mean from 5 data points (n = 5). (**e**) Real time PCR results of expression of rhBMP-2-mediated genes and BMPRs genes on different samples. Values are shown as mean ± standard error of the mean from 5 data points (n = 5). ^*^p < 0.05, compared with the corresponding surfaces without rhBMP-2; ^#^*p* < 0.05, compared with HAP/rhBMP-2; ^@^*p* < 0.05, compared with rhBMP-2 solution.

The adsorption stability of rhBMP-2 on the HAP and Mg-HAP surfaces was shown in Fig. 4a. It was found that both the HAP/rhBMP-2 and Mg-HAP/rhBMP-2 samples exhibited no significant difference (p > 0.05) in the adsorption stability of rhBMP-2: an initial burst release within first 24 h and a gradual release after that. Notably, more than 85% of rhBMP-2 remained on the HAP and Mg-HAP surfaces, which indicated that the growth factors were tightly adsorbed and had good adsorption stability. It can be inferred that the Mg-HAP/rhBMP-2 was acted as a complex communicating with cells, which was also supported by an ALP study (Fig. S5, Supporting Information) suggesting Mg^2+^ and released rhBMP-2 hardly induced ALP activity but the complex did. The osteogenetic bioactivity of adsorbed rhBMP-2 was determined by quantitating the ALP expressions of C2C12 cells (Fig. 4b). It was clearly found that the HAP and Mg-HAP samples (as negative control) exhibited little ALP expression. At days 3 and 5, the Mg-HAP/rhBMP-2 sample induced significantly (p < 0.05) increased ALP activity of C2C12 cells compared to the HAP/rhBMP-2 sample. Importantly, the ALP expression normalized to rhBMP-2 amount (data not shown) was significantly higher (p < 0.05) for C2C12 cells cultured on the Mg-HAP/rhBMP-2 surface than on the HAP/rhBMP-2 surface. Intriguingly, the bioactivity of adsorbed rhBMP-2 on the Mg-HAP surface was notably greater (p < 0.05) than the theoretical positive control, most likely due to the contributing synergistic effects of the nanostructured Mg-HAP surface and desirable conformation of adsorbed rhBMP-2. In addition, the enhanced bioactivity of rhBMP-2 on the Mg-HAP surface was also evidenced by the Western Blot and Quantitative real time PCR results (Fig. 4c–e) showing significantly (p < 0.05) promoted p-Smad 1/5/8 and expression of Id1, Runx2, and OCN of C2C12 cells on the Mg-HAP/rhBMP-2 group.

### Expression of BMPRs in C2C12 cells

**Figure 5:**
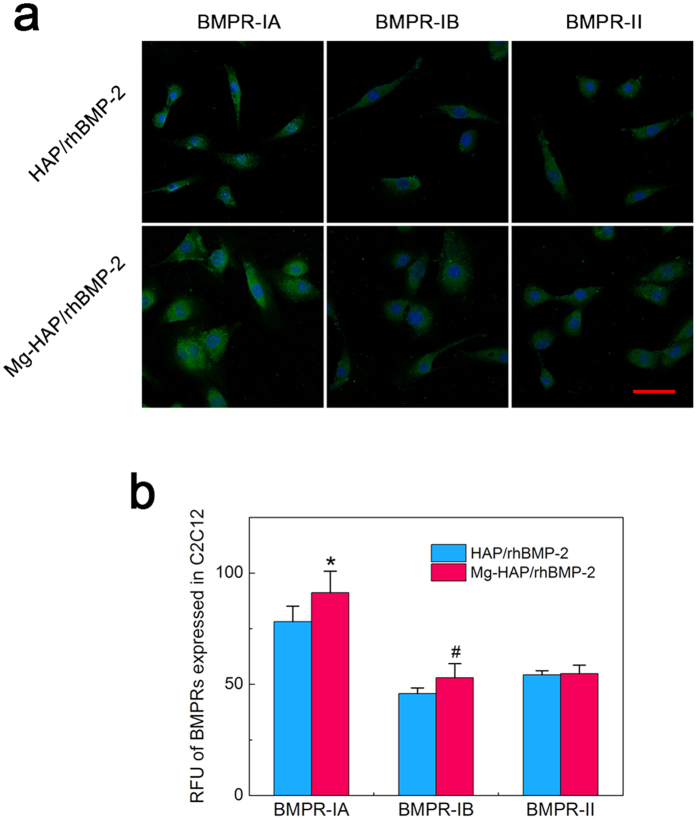
Expression of BMPRs (BMPR-IA, BMPR-IB, and BMPR-II) on cellular surface of C2C12 cells cultured on the HAP/rhBMP-2 and Mg-HAP/rhBMP-2 surfaces. (**a**) Fluorescence images of BMPRs (green dots, respectively) and cell nucleus (blue) of C2C12 cells cultured on respective surfaces after 12 h of incubation. Scale bar is 50 μm. (**b**) The RFU (relative fluorescence units) of BMPRs expressed in C2C12 cells extracted from fluorescence pictures of BMPRs. Values are shown as mean ± standard error of the mean from 50 data points (n = 50). *p < 0.05, #p < 0.05, compare with the HAP/rhBMP-2 surface.

To further explore Mg mediating rhBMP-2/BMPRs interactions, the expression of BMPRs on cellular surface of C2C12 cells cultured on the rhBMP-2-adsorbed surfaces was investigated by an immunofluorescence staining of the BMPR-IA, BMPR-IB, and BMPR-II respectively. As shown in Fig. 5a, the expressed BMPRs are uniformly distributed on the cellular surface of C2C12 cells cultured on the HAP/rhBMP-2 and Mg-HAP/rhBMP-2 surfaces. Importantly, significant larger (p < 0.05) BMPR-IA and BMPR-IB clusters and comparable BMPR-II clusters are found on the C2C12 cells seeded on the Mg-HAP/rhBMP-2 sample than on the HAP/rhBMP-2 sample. In addition, the RFU of BMPRs (Fig. 5b) revealed that the BMPR-IA and BMPR-IB were more expressed in the C2C12 cells cultured on the Mg-HAP/rhBMP-2 surface, which well corresponds to the QCM-D studies and the immunofluorescence staining experiments (Table 2 and Fig. 3c–e). In addition, Quantitative real time PCR was applied to identify the expression of BMPRs at the transcriptional level. As shown in Fig. 4e, the Mg-HAP/rhBMP-2 surface significantly promoted the cellular expression of BMPR-IA, ActR-I and ActR-IIB. Considering that the low binding availability of ActR-I and ActR-IIB, the ameliorated bioactivity of rhBMP-2 on the Mg-HAP/rhBMP-2 group is ascribed to the up-regulated BMPR-IA-binding availability and promoted BMPR-IA expression in C2C12 cells.

### Cell adhesion, focal adhesion, and cytoskeleton organization

**Figure 6:**
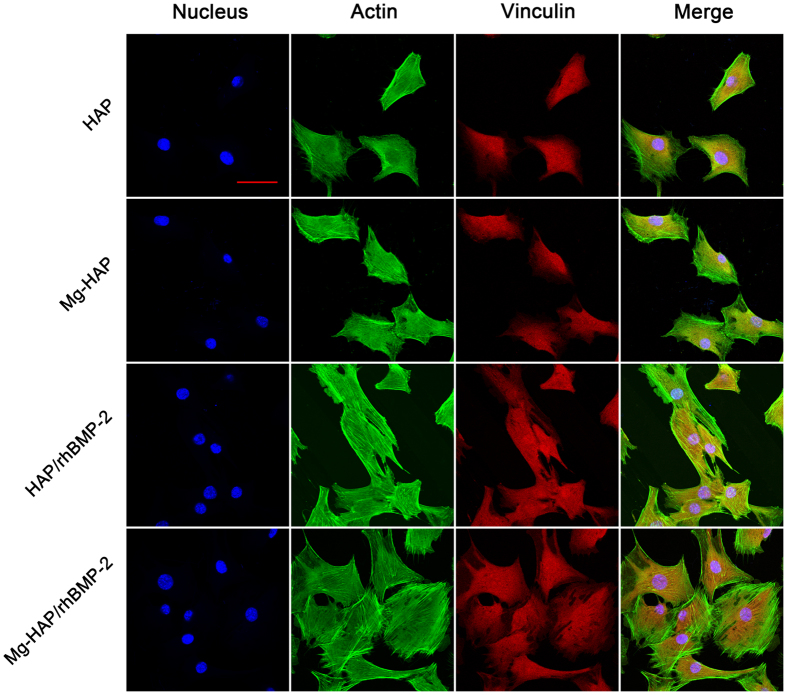
Fluorescence images of actin (cytoskeleton, green), vinculin (focal contact, red), cell nucleus (blue), and merged images of C2C12 cells cultured on respective surfaces after 12h of incubation. Scale bar is 50 μm.

The cellular morphology, focal adhesion, and cytoskeleton organization of C2C12 cells on the respective surfaces were investigated with an immunofluorescence staining of the actin, vinculin, and nucleus (Fig. 6). It can be seen that C2C12 cells were well spread and had intimate contact with the surfaces, especially for the HAP/rhBMP-2 and Mg-HAP/rhBMP-2 samples. The cell amounts were obviously larger (p < 0.05) on the HAP/rhBMP-2 and Mg-HAP/rhBMP-2 surfaces as compared to that on the respective HAP and Mg-HAP surfaces. Moreover, C2C12 cells cultured on the Mg-HAP/rhBMP-2 surface showed a better spread morphology and a well-organized actin cytoskeleton when compare to those of the HAP/rhBMP-2 surface. The vinculins (visible as red background) were present throughout the cytoplasm of all C2C12 cells, suggesting large focal contacts between cells and respective surfaces. It is evident that greater focal adhesion was formed on the Mg-HAP/rhBMP-2 surface as compared to the HAP/rhBMP-2 surface. To further investigate the cellular attachment, assessments of the cell-surface contact area and cellular perimeter were performed (Fig. S6, Supporting Information). It was found that C2C12 cells cultured on the HAP/rhBMP-2 and Mg-HAP/rhBMP-2 surfaces had larger contact area than that on the HAP and Mg-HAP surfaces, respectively. Importantly, a significant (p < 0.05) increased contact area was achieved on the Mg-HAP and Mg-HAP/rhBMP-2 surfaces as compared to the HAP and HAP/rhBMP-2 surfaces, respectively. It is noteworthy that the data of cellular perimeter exhibited similar results to those of the cell-surface contact area. Similar to some recent studies^13^,^21^, these findings suggested that rhBMP-2 adsorbed on the HAP and Mg-HAP surfaces, especially on the Mg-HAP surface, can significantly improve C2C12 adhesion.

### Molecular dynamic simulation

**Figure 7:**
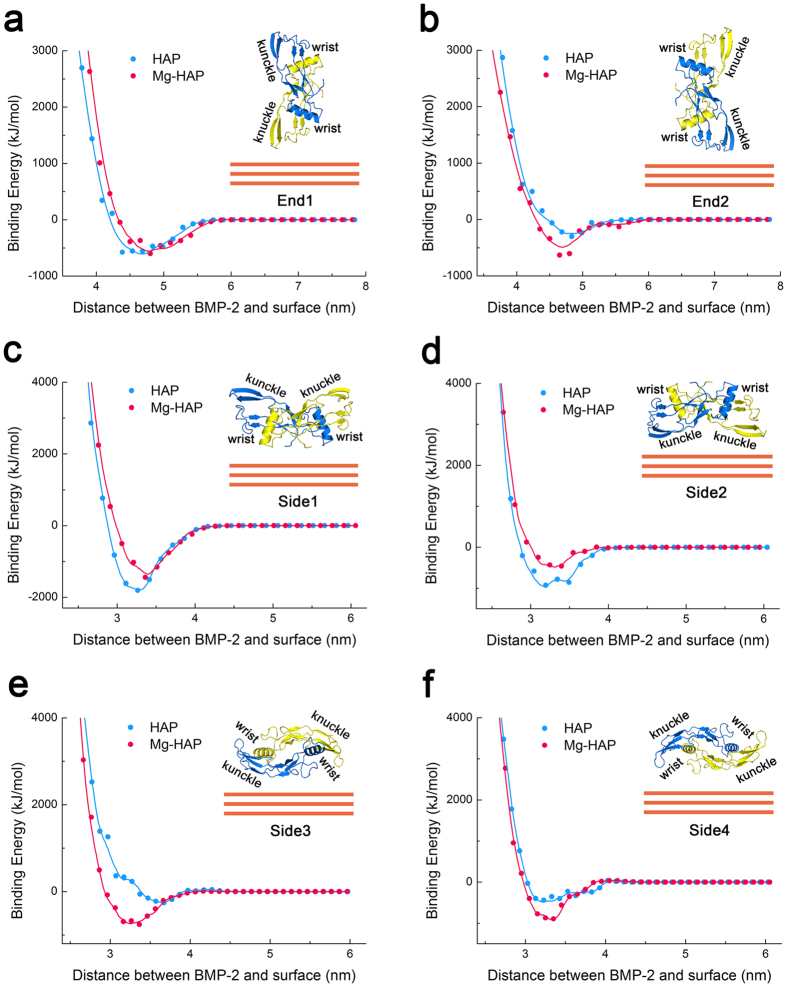
Binding energy against the distance between BMP-2 (centre of mass) and surface (HAP and Mg-HAP) for the End1 (**a**) End2 (**b**) Side1 (**c**) Side2 (**d**) Side3 (**e**) and Side4 (**f**) orientations. Inserts are illustrations of respective configurations of BMP-2 (with wrist and knuckle epitopes noted) towards surface.

In order to shed light on the dynamic adsorption of BMP-2 on the HAP and Mg-HAP surfaces at atomic level, we performed combined MD and SMD simulations. The binding energy against the distance between BMP-2 and the surface in the SMD process is provided in Fig. 7. In the End1 orientation, there was no significant difference (p > 0.05) between the binding energies for the HAP and Mg-HAP surfaces. In the Side1 and Side2 orientations, it was found that the binding energy for the HAP surface was lower than that for the Mg-HAP surface. However, in the End2, Side3, and Side4 orientations, a remarkably lower (p < 0.05) binding energy was observed on the Mg-HAP surface compared to that on the HAP surface. These findings suggest that BMP-2 adsorbed in different orientations towards the HAP and Mg-HAP surfaces exhibited distinct interactions with these surfaces. Moreover, we recorded the minimum binding energy and the separation distance of the adsorption state (Table S1 and Table S2, Supporting Information). Basically, the separation distances between BMP-2 and the surface for side-on orientations were notably shorter (p < 0.05) than that for end-on orientations.

The radius of gyrate (Rg) of BMP-2 with respect to simulation time is shown in Fig. S7 (Supporting Information). It was also found that the Rg of BMP-2 for the whole process was significantly lower (p < 0.05) in all end-on orientations, while it was notably higher (p < 0.05) in all side-on orientations. This finding suggests that the configuration of BMP-2 was partly folded during the adsorption process in the end-on orientations, while it was partly loosened during the adsorption process in the side-on orientations. Moreover, various Rg shifts (Table 3) indicated that BMP-2 molecules were folded or loosened at different extents on the HAP and Mg-HAP models as compared to the initial configuration. In addition, the profile of root mean square deviation (RMSD) of BMP-2 against the simulation time is shown in Fig. S8 (Supporting Information). All the curves exhibited a similar trend: a linear increase of RMSD from 0 to 600 ps, a rapid up-regulation of RMSD from 600 to 750 ps, and a slight rise of RMSD in the MD procedure (750 to 2750 ps). The RMSDs of BMP-2 on the HAP and Mg-HAP models varied slightly.

**Figure 8:**
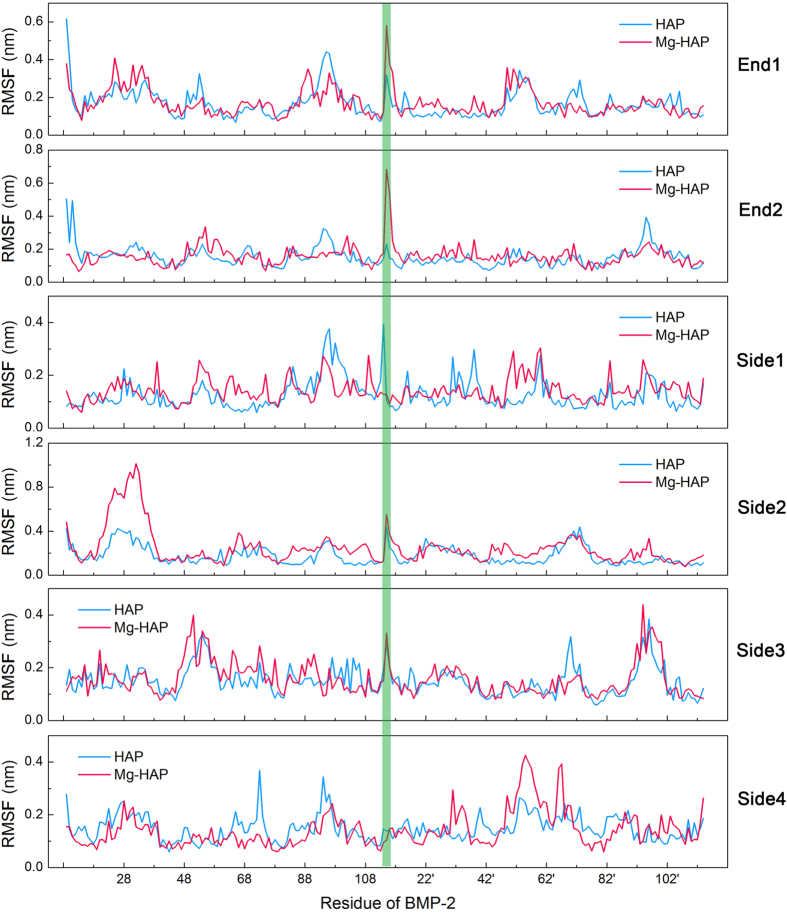
RMSF of residues of BMP-2 (with 6 configurations) on the HAP and Mg-HAP surfaces during the adsorption process. BMP-2 is composed by 2 monomers. Monomer I is composed by residues Arg9 to Arg114, and monomer II is composed by residues Arg9′ to Arg 114′.

The root mean square fluctuation (RMSF) of residues of BMP-2 was also obtained in the whole adsorption process (Fig. 8). At first glance, the values of RMSF are varied with orientation. As expected, the values of RMSF are also varied on HAP and Mg-HAP surfaces in a same orientation of BMP-2. Moreover, the residues which exhibited a high value of RMSF (>0.25 nm) are collected in Table S3 (Supporting Information). It was found that a rash of residues showed a high value of RMSF for BMP-2 adsorbed on the HAP and Mg-HAP surface in the Side2 orientation. Regardless of the orientation of BMP-2, significantly more residues with a high value of RMSF on the Mg-HAP surface were found than that on the HAP surface. In particular, we found that it was extremely high (>0.6 nm) for the RMSFs of residues Phe23-Ala34 of BMP-2 in the Side2 orientation on the Mg-HAP surface, which might lead to denaturation of the adsorbed protein. In addition, the values of RMSF of cysteine-knots of BMP-2 were relative low (Fig. S9, Supporting Information). It can be found that the cysteine-knots of BMP-2 showed different stabilities in 6 orientations both on the HAP and Mg-HAP surfaces. Together, the results indicated that the cysteine-knots of BMP-2 on the Mg-HAP surface were more stable than that on the HAP surface.

## Discussion

Adsorption of proteins onto surfaces of inorganic material is a crucial topic in the field of biomaterials, biomedicine, biosensors, and cell biology^16^,^17^,^19^. It is reported that proteins from the extracellular matrix (ECM) firstly attach to the surface of an implanted material, and then sensitively mediate the cellular adhesion^12^,^13^,^17^. The biological processes of adsorption are proved to have a strong dependence on the interaction between the protein and material surface. The protein-surface interactions can correspond to the distinct surface properties (e.g. charge^16^, chemistry^17^, topography^18^, and hydrophilicity^19^), natures of proteins^12^, and the surrounding environment (e.g. pH^7^ and ions^19^). If the biological performance of the sequestrated protein can be preserved or even enhanced, the surface will be highly desirable in regenerative medicine and tissue engineering as novel functionalities are integrated into the materials^10^,^26^. To that end, in the present study we both experimentally and numerically investigated the adsorption, BMPRs-recruitment, and bioactivity of rhBMP-2 on the HAP and Mg-HAP surfaces. With these typical matrixes as material models, the results indicated that the BMPR-IA binding capacity and bioactivity of adsorbed rhBMP-2 were significantly up-regulated on the Mg-HAP surface compared to the HAP surface, even though the adsorption amount of rhBMP-2 was slightly decreased. Moreover, combined MD/SMD simulations revealed that the BMP-2 on the Mg-HAP surface largely preserved the active protein structure evidenced by more stable cysteine-knots as compared to the HAP surface. Collectively, this contribution could provide valuable guidance for the future design and fabrication of BMP-2-based bone scaffolds/matrixes.

It is well-acknowledged that the primary interactions between proteins and substrate surfaces, especially at the initial adsorption process, mainly depend on hydrophobic interaction, electrostatic interaction, hydrogen bond, surface topography, and surface chemistry^14^,^16^,^17^,^18^,^19^. The hydrophobic interaction, electrostatic interaction, and surface topography were not significantly different between the HAP/rhBMP-2 and Mg-HAP/rhBMP-2 samples, given that the HAP and Mg-HAP surfaces exhibited similar water contact angles, zeta potentials, and RMS values (Table 1). For the hydrogen bond, recent studies have demonstrated that it is closely related with the hydrophobic interaction and electrostatic interaction in the protein-surface interactions^19^,^36^,^40^. Thus, the hydrogen bonds were also not significantly different between the HAP/rhBMP-2 and Mg-HAP/rhBMP-2 samples. Actually, in this study the surface chemistry was the predominant reason for the distinct interaction between the HAP/rhBMP-2 and Mg-HAP/rhBMP-2 samples. With a sparse Mg presenting in the Mg-HAP surface, the Van der Waals force was shifted as compared to the HAP surface. This discrepancy was confirmed to lead the variation of conformation and/or orientation of rhBMP-2 on the HAP and Mg-HAP surfaces, which ultimately influenced the bioactivity of the adsorbed protein.

It can be inferred that the rhBMP-2 molecules possessed better bioactivity on the Mg-HAP surface than on the HAP surface. As a bone growth factor, rhBMP-2 exerts its signal function mainly through oligomerizing BMPR-IA/B and BMPR-II receptors serine/threonine kinases on the cellular membranes^41^,^42^. Moreover, the recognition of BMPR-IA to rhBMP-2 is demonstrated as a pivotal role for signal transduction^43^. The better bioactivity of Mg-HAP/rhBMP-2 is ascribed to the higher expression of BMPR-IA in C2C12 cells (Figs 4e and 5) and the greater BMPR-IA/B-recognition of the adsorbed rhBMP-2 (Table 2 and Fig. 3c–e). These promoted BMPR-IA-expression and BMPR-IA/B-recognition are closely related to the loosened configuration of adsorbed rhBMP-2 on the Mg-HAP surface, which is well supported by the values of (ΔD/Δf)_rhBMP-2_. In conclusion, the Mg-HAP surface induced favorable configuration of rhBMP-2, up-regulated rhBMP-2/BMPR-IA/B recognition, and enhanced expression of BMPR-IA in C2C12 cells. In addition, the cellular attachment study also supported that the rhBMP-2 on the Mg-HAP surface had a better bio-compatibility and bio-performance, which was evidenced by the C2C12 cells cultured on the rhBMP-2-adsorbed Mg-HAP surface having a well-organized cytoskeleton (Fig. 6 and Fig. S3, Supporting Information). Moreover, the priority of Mg-HAP/rhBMP-2 to HAP/rhBMP-2 was also confirmed by an ALP study using BMSCs (Fig. S5, Supporting Information).

It is interesting and necessary to investigate various orientations of BMP-2 for adsorbing on a surface, as some of them are highly favored^39^,^40^. In the present study, we selected 6 typical orientations of BMP-2 as the objectives of MD/SMD simulations. In recent years, several studies^36^,^38^ have investigated the adsorption of different orientations of BMP-2 with molecular models, but they have not explored the difference between the various orientations and the most favorable orientation. Here, with the curve of binding energy, it was shown that the Side1 orientation of BMP-2 was the most preferred orientation and had the strongest interaction with both the HAP and Mg-HAP surfaces (Fig. 7 and Table S1, Supporting Information). Considering the random orientations of BMP-2 in solution and binding energy, there were also some other possible orientations (especially Side2, Side3, and Side4) of BMP-2 that adsorbed on the HAP and Mg-HAP surfaces. For the HAP surface, the majority of BMP-2 was adsorbed in the Side1 and Side2 orientations. For the Mg-HAP surface, though the most favorable orientation is still Side1, a considerable amount of BMP-2 was adsorbed in the Side3 and Side4 orientations.

It is believed that MD and SMD simulations have greatly advanced knowledge of protein structures^37^,^38^, as they can predict many parameters of protein configuration. Among the parameters, Rg and RMSD are the mostly calculated items for evaluating conformational changes of proteins. Here, in the End1 and End2 orientations, it can be inferred that the adsorbed BMP-2 was folded on both HAP and Mg-HAP surfaces (Table 3). Further, a more highly folded conformation of BMP-2 was achieved on the Mg-HAP surface than that on the HAP surface. In all side on orientations, it was found that the adsorbed BMP-2 was loosened on both HAP and Mg-HAP surfaces (Table 3). Specifically, in the Side1 and Side3 orientations, it was found that the adsorbed BMP-2 was in a less loosened conformation on the Mg-HAP surface than on the HAP surface; in the Side2 and Side4 orientations, we found that the adsorbed BMP-2 was in a more loosened conformation on the Mg-HAP surface as compared to that on the HAP surface. As is widely accepted, a slightly loosened configuration of protein could expose its active spots for ligand-receptor interaction and thus up-regulate the bioactivity of protein^11^,^12^,^13^,^44^. Therefore, it can be inferred that the bioactivities of BMP-2 with all end on orientations were decreased, while with all side on orientations were increased, for both the HAP and Mg-HAP surfaces. It seems that this conclusion has a contradiction to the experiment results that the ALP activities of BMP-2 on both the HAP and Mg-HAP surfaces were higher than those of the theoretical positive control (Fig. 4b). However, considering that the preferred orientations for BMP-2 adsorbed on the HAP and Mg-HAP surfaces, it is reasonable and clear that the bioactivity of adsorbed BMP-2 is enhanced on both surfaces, and it is higher for the Mg-HAP surfaces.

As is known to all, a globe protein is composed of a hydrophobic core and some hydrophilic residues^16^,^44^, which are important to stabilize the whole structure. However, BMP-2 does not possess a hydrophobic core, thus 7 cysteine-knots of BMP-2 play core roles in maintaining functional structure of the protein^41^,^44^. Some recent studies^23^,^25^,^26^,^44^ have suggested that the maintenance of integrity of cysteine-knots can preserve a high bioactivity of BMP-2, and the depletion of cysteine-knots cause a denaturation of BMP-2. Therefore, the stability of cysteine-knots could be utilized as an additional perspective for assaying the bioactivity of adsorbed BMP-2. To further explore the collections between the bioactivity and structures of BMP-2, we evaluated the stability of cysteine-knots of BMP-2. As the results indicated, the cysteine-knots located in different monomers of BMP-2 exhibited a distinguished stability (Fig. S9, Supporting Information). Collectively, in general we found that cysteine-knots of BMP-2 on the Mg-HAP surface were more stable as compared to the HAP surface. It is believed that the existence of Mg on the Mg-HAP surface restrained the movement of cysteine-knots of BMP-2, and thus preserved the higher bioactivity of BMP-2 compared to the HAP surface.

**Figure 9:**
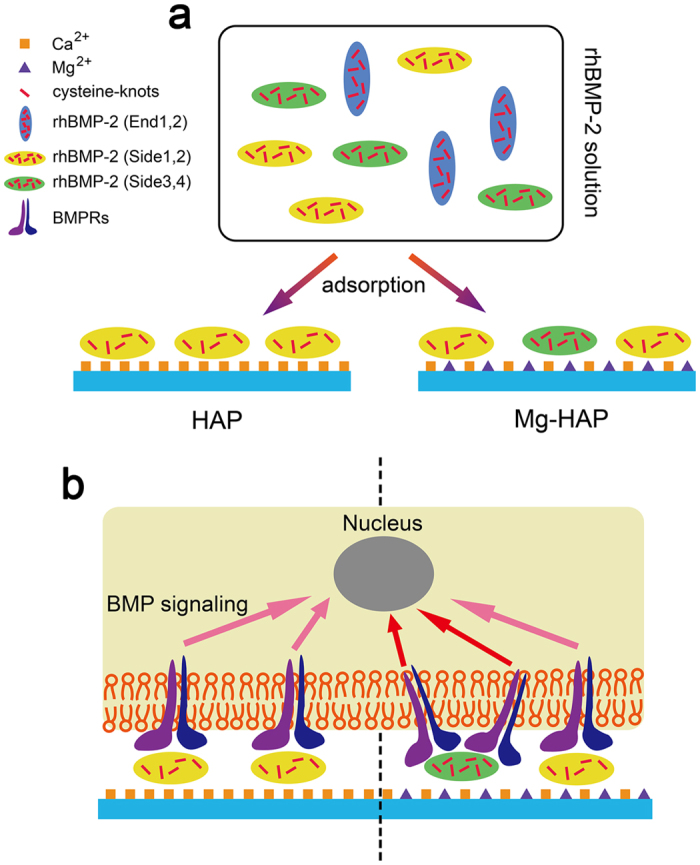
Schematic diagram of the adsorption of rhBMP-2 and recognition of BMPRs to rhBMP-2 on the HAP and Mg-HAP surfaces. (**a**) For the HAP surface, Side1 and Side2 are the most favorable orientations for the adsorption of rhBMP-2. For the Mg-HAP surface, Side1 and Side2 (especially Side1) are the most favorable orientation for the adsorption of rhBMP-2, while a considerable amount of rhBMP-2 is adsorbed in Side3 and Side4 orientations. (**b**) The rhBMP-2 on the Mg-HAP surface exhibited a greater BMPRs-recognition than that on the HAP surface, which is ascribed to the slightly loosened conformation and more stable cysteine-knots. The higher BMPRs-recognition on the Mg-HAP surface induced a stronger BMP signaling than that on the HAP surface.

In summary, the enhanced bioactivity of rhBMP-2 on the Mg-HAP surface should be attributed to the synergistic effect of the preferred orientation, loosened conformation, and stable cysteine-knots (Fig. 9). The present study systematically investigated the differences of the adsorption of rhBMP-2 on the HAP and Mg-HAP surfaces and the involved mechanism. Moreover, it is for the first time it has been demonstrated that the BMP-2 adsorbed on the Mg-HAP surface has better stability of cysteine-knots than that on the HAP surface. All acquired knowledge in this study will contribute to a better understanding of protein-surface interactions and up-regulation of bioactivity of BMP-2.

## Conclusion

With experimental and numerical assays, we have demonstrated that the recruitment of BMPR-IA and bioactivity of BMP-2 were remarkably increased on the Mg-HAP surface compared to those on the HAP surface. The presence of a low amount of Mg on the Mg-HAP surface not only induced a slightly loosened conformation of BMP-2, but also maintained the integrity of cysteine-knots of the adsorbed BMP-2. The clear advantages of the Mg-HAP surface for loading BMP-2 highlight the potential of Mg-HAP biomaterials in being used as bone regeneration implants/scaffolds.

## Materials and methods

### Materials

Ca(NO_3_)_2_⋅4H_2_O and (NH_4_)_2_HPO_4_ were purchased from Sinopharm Chemical Reagent Co., Ltd. Shanghai, China. NH_3_⋅H_2_O and Mg(NO_3_)_2_⋅6H_2_O were purchased from Shanghai Lingfeng Chemical Regent Co., Ltd. Shanghai, China. Gold-coated QCM-D sensors (QSX 301) were purchased from Q-Sense AB, Bioline Scientific, Sweden. *Escherichia coli*-derived rhBMP-2 (carrier-free, >95% purity) was obtained from Shanghai Rebone Biomaterials Co., Ltd. Shanghai, China. Recombinant Human BMPRs (BMPR-IA, BMPR-IB, BMPR-II, ActR-I, ActR-II, and ActR-IIB, expressed in mouse NSO cells) and Human BMP-2 ELISA Kit were obtained from R&D Systems, Inc. Minneapolis, USA. FluoroTag FITC conjugation Kit, TRITC conjugation Kit, mouse monoclonal anti-vinculin antibody, TRITC-conjugated goat anti-mouse IgG antibody, and FITC-conjugated phalloidin were purchased from Sigma-Aldrich, St Louis, USA. DMEM, FBS, 0.25% Trypsin-EDTA, and PBS were obtained from GIBCO, Grand Island, USA. BCA assay kit, ALP assay kit and DAPI were purchased from Beyotime, Biotech. Jiangsu, China. Rabbit polyclonal anti-human/mouse BMPR-IA, BMPR-IB, and BMPR-II antibody, FITC-conjugated goat anti-rabbit IgG antibody, and goat polyclonal HRP-conjugated anti-rabbit IgG were purchased from Abcam, Cambridge, UK. Rabbit polyclonal anti-Smad1/5/8, anti-phospho-Smad1/5/8, and anti-GAPDH were purchased from Cell Signaling Technology, Beverly, USA. Trizol reagent, PrimeScript RT reagent Kit, and SYBR Premix Ex Taq were purchased from Takara, Tokyo, Japan.

### Fabrication and characterization

#### Preparation of HAP and Mg-HAP nanoparticles

Colloidal HAP nanocrystals were prepared by a microwave method. In brief, 0.040 mol of Ca(NO_3_)_2_⋅4H_2_O was dissolved in 200 mL of deionized-distilled water free of CO_2_. 0.024 mol of (NH_4_)_2_HPO_4_ was dissolved in 120 mL of deionized-distilled water free of CO_2_. After freezing in a −20 °C desk refrigerator for 5 min, the two above-mentioned solutions were mixed with mild stirring, and maintained the pH around 10.5 with NH_3_⋅H_2_O during the procedure. The mixed solution was then set in a microwave synthesis reactor at 200 W and 60 °C for 30 min. The resultant suspension was centrifuged at 6000 g for 10 min, and washed 3 times with deionized water and ethanol (in that order) and finally dried at 70 °C in an air oven for 24 h. To obtain the Mg-HAP nanocrystals, 0.040 mol of Ca(NO_3_)_2_⋅4H_2_O was substituted by 0.002 mol of Mg(NO_3_)_2_⋅4H_2_O and 0.038 mol of Ca(NO_3_)_2_⋅4H_2_O in the above procedure.

#### Characterization of HAP and Mg-HAP nanoparticles

Phase compositions and crystal sizes of the nanocrystals were characterized by XRD (Rigaku RU-200). Surface charges of HAP and Mg-HAP nanocrystals in deionized water were determined by dynamic light scattering using a Zetasizer Nano Series. Surface morphologies of the nanoparticles were observed by TEM (JEOL JEM-2100) with SAED. Functional groups of HAP and Mg-HAP nanoparticles were analyzed by FTIR (Nicolet Nexus 670). Chemical compositions of the nanoparticles were quantified by EDS (QUANTAX 400-30).

#### Preparation of the HAP and Mg-HAP surfaces

We employed an EPD method to fabricate an ultra-thin layer of the HAP and Mg-HAP coatings on QCM-D sensors, respectively. Briefly, a platinum gauze electrode (1.5 × 1.5 cm) served as the anode, and the QCM-D sensor (gold-plated) served as the cathode. For the HAP coating, 1 wt% of the HAP suspension in ethanol was used as electrolyte. And, 100 V/cm DC voltage was applied for 3 min to prepare the HAP coating on the QCM-D sensor. For the Mg-HAP coating, 1 wt% of Mg-HAP suspension in ethanol was used as electrolyte in the above procedure. After the deposition, the coated QCM-D sensors were conducted with an ultrasonic treatment (40 kHz, 75W, 0.5 min) and dried with nitrogen. For cellular assessments, the coated QCM-D sensors were sterilized using an autoclave at 120 °C for 30 min.

#### Characterization of the HAP and Mg-HAP surfaces

Surface morphologies and elemental distributions of the HAP and Mg-HAP surfaces were investigated by SEM coupled with X-ray energy dispersive spectrometry (SEM-EDS, Hitachi S-4800). Surface roughness of the prepared surfaces was analyzed in air by the AFM (Shimadzu SPM-9500). Surface charges of the HAP and Mg-HAP surfaces in deionized water were determined using SurPASS (AntonPaar). Surface hydrophilicities of the HAP and Mg-HAP surfaces were analyzed at 22 °C in air by a sessile drop method of distilled water (approximately 0.02 mL) with automatic contact angle meter (Kyowa Interface Science CA-W200). Thicknesses of the HAP and Mg-HAP coatings were examined with a spectral ellipsometer (UVISEL/VIS Jobin Yvon). Concentrations of ions released from the HAP and Mg-HAP surfaces at 37 °C for 1, 3, and 7 days were examined by inductively coupled plasma optical emission spectroscopy (ICP-OES, Optima 8300).

### RhBMP-2 adsorption and BMPRs binding capacity

#### QCM-D study

The surface adsorption of rhBMP-2 and the ensuing BMPRs (BMPR-IA, BMPR-IB, BMPR-II, ActR-I, ActR-II, and ActR-IIB) binding to the adsorbed rhBMP-2 layer were monitored by the well-used QCM-D technique. According to the simple Sauerbrey equation (shown below)^11^, a thin non-dissipative layer with no slip condition, the adsorbed mass, Δ*m* (ng/cm^2^), is directly proportional to the frequency shifts, Δ*f* (Hz).


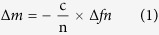


where C (17.7 ng·cm^2^ Hz^−1^ for a 5 MHz crystal) is the mass sensitivity constant, n (1, 3, 5, …) is the overtone number, and Δ*f*n is the frequency shifts of the nth overtone.

The BMPRs binding to the rhBMP-2 adsorbed surfaces results in a Δ*f* and the additional mass was analyzed as an estimate of “BMPRs-binding availability” with Sauerbrey equation by the following equation^11^:


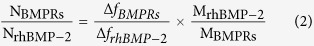


where N_rhBMP-2_ is the number of rhBMP-2 molecules in the protein layer, N_BMPRs_ is the number of BMPRs bound to the rhBMP-2 layer, Δ*f*_*rhBMP-2*_ and Δ*f*_*BMPRs*_ are the respective frequency changes, and M_rhBMP-2_ = 26 kDa, M_BMPR-IA_ = 55 kDa, M_BMPR-IB_ = 55 kDa, M_BMPR-II_ = 75 kDa, M_ActR-I_ = 39 kDa, M_ActR-II_ = 40 kDa, and M_ActR-IIB_ = 41 kDa are the respective molecular weights.

The monitoring of the adsorption of rhBMP-2 on the prepared surfaces and the subsequent binding of BMPRs to rhBMP-2 adsorbed surfaces were conducted as follows: the flow rate (20 μL/min) and temperature (37 °C) were kept constant throughout all measurements. First, pure PBS was injected into the QCM-D chamber to stabilize the baseline for 10 min. Then, the rhBMP-2 (50 μg/mL) was introduced into the QCM-D chamber, and the QCM-D measurement parameters were stabilized for 30 min. After that, the PBS was used to rinse the saturate adsorbed protein layer for 5 min. At approximately t = 45 min, the BMPRs (respectively, 10 μg/mL) were introduced into the QCM-D chamber, and the QCM-D measurement parameters were stabilized for 30 min, following which the PBS was used to rinse the layer again for 5 min. Here, the uses of concentration of rhBMP-2 and BMPRs followed literature^11^,^12^,^24^ and manufacturer’s instructions. In addition, various concentrations of rhBMP-2 were utilized to explore the equilibrium binding constant and maximum binding capability.

#### Immunofluorescence assay

An immunofluorescence assay was performed for further identifying the adsorption of rhBMP-2 on the HAP and Mg-HAP surfaces and subsequent binding of BMPRs to the adsorbed rhBMP-2. FITC was reacted with the primary amines of rhBMP-2 according to the protocol recommended by the manufacturer. TRITC was reacted with the primary amines of BMPRs according to the protocol of FluoroTag FITC conjugation kit recommended by Sigma-Aldrich. The FITC-conjugated rhBMP-2 was adsorbed on the HAP and Mg-HAP surfaces as the same procedure in the QCM-D measurements. Briefly, the prepared surfaces were immersed into 0.2 mL FITC-labeled rhBMP-2 (50 μg/mL) for 30 min at room temperature. Then the FITC-labeled rhBMP-2 solutions were removed and the substrates were washed twice with PBS to rule out the unadsorbed rhBMP-2. Following this, the rhBMP-2-adsorbed surfaces were blocked with 5% BSA at room temperature for 2 h, and then incubated with TRITC-conjugated BMPRs for 2h at 4 °C. The samples were kept in the dark at 4 °C until the substrates were observed by confocal laser scanning microscopy (CLSM, Nikon A1). Images at 600X magnification were acquired at 10 randomly chosen fields on 5 independent samples for each surface. BMPRs-TRITC and rhBMP-2-FITC were also applied in QCM-D experiments to indentify the binding availability of labeled BMPRs and rhBMP-2.

### Adsorption stability of rhBMP-2

The adsorption stability of growth factor from the rhBMP-2-adsorbed HAP and Mg-HAP surfaces were examined with a Human BMP-2 ELISA Kit over a period of 5 days. Similar to the adsorption processes of QCM-D experiments, rhBMP-2 was adsorbed on the HAP and Mg-HAP surfaces by an immersion method. In brief, the HAP and Mg-HAP surfaces were immersed into 0.2 mL rhBMP-2 (50 μg/mL) for 30 min at 37 °C, respectively. Then, the rhBMP-2 solutions were removed and the rhBMP-2-adsorbed surfaces were washed twice with PBS to rule out the surplus rhBMP-2. Following this, the rhBMP-2-adsorbed surfaces were put into 24-well plates containing 2 mL PBS and incubated at 37 °C, with constant agitation at 15 rpm. At the end of each time point, the release medium was collected (200 μL) and replenished with an equal amount of fresh PBS solution. All samples were frozen and stored at −80 °C until analysis. Profiles of stability of rhBMP-2 were calculated in terms of the remained percentage of adsorbed rhBMP-2 (%, w/w) with incubation time. The measurements were performed in triplicate for each time point.

### BMPRs expression, Bioactivity, and cell attachments

#### Cell culture

C2C12 cells, purchased from the American Type Culture Collection (ATCC), were cultured in 37.5 cm^2^ flasks with DMEM containing 10% FBS, antibiotics (100 U/mL penicillin-G and 100 mg/mL streptomycin) at a humidified atmosphere of 5% CO_2_/95% air until confluence. After being detached with 0.5 mL 0.25% trypsin-EDTA, cells were suspended in normal culture medium. The cell density was calculated and used at the desired density in later assays. Rat BMSCs were isolated and cultured based on protocols from previous studies^22^,^33^. BMSCs of the fourth to sixth passage were used in the present study.

#### BMPRs expression

To further explore Mg affecting rhBMP-2/BMPRs interactions, we examined the expression of BMPRs in C2C12 cells cultured on the HAP/rhBMP-2 and Mg-HAP/rhBMP-2 surfaces. Briefly, rhBMP-2 was adsorbed on the HAP and Mg-HAP surfaces as discussed in the adsorption stability study. Then, the C2C12 cells were seeded on these surfaces at a density of 1 × 10^4^ cells/well (24-well plate). After 12h incubation, the cells were fixated with 10% neutral PBS buffered formalin (10 min) and then incubated with 0.1% Triton X-100 for 1h to permeabilize the cells. The BMPRs on cellular surface were stained with rabbit polyclonal anti-human/mouse BMPR-IA, BMPR-IB and BMPR-II antibodies and FITC-conjugated goat anti-rabbit IgG antibodies according to the manufacturer’s instructions. DAPI was used to stain the cell nucleus at a concentration of 0.5 μM DAPI (200 μL). Then, the stained cells were investigated by CLSM with 600× magnification at 10 randomly chosen fields on 5 independent samples.

#### Bioactivity of the adsorbed rhBMP-2

To explore the bioactivity of adsorbed rhBMP-2, we measured the ALP activity of C2C12 cells induced by the rhBMP-2 adsorbed on the HAP and Mg-HAP surfaces. In brief, rhBMP-2 was adsorbed on the HAP and Mg-HAP surfaces as discussed in the adsorption stability study. To prepare the negative control experiments, the rhBMP-2-unadsorbed surfaces were achieved by immersing the substrates into 0.5 mL pure PBS solution. After removing the immersion liquid, the C2C12 cells were seeded on the aforementioned surfaces at a density of 4 × 10^4^ cells per well (24-well plate). Culture medium with a similar content of rhBMP-2, corresponding to that calculated from the QCM-D study, was applied as the positive control. After 1, 3, and 5 days of incubation, ALP activity was measured according to standard protocols. The absorbance of ALP was quantified at the wavelength of 405 nm using a microplate reader (SPECTRAmax 384, Molecular Devices, USA). ALP activity was expressed as 405 nm OD value per total protein per min (OD/mg protein/min). In addition, BMSCs were also utilized in the ALP study according to protocols of previous study^33^.

#### Western blot study

Phosphorylation of Smad1/5/8, a short-term effect of BMP signaling, was semi-quantitatively analyzed by the western blot assay. Briefly, rhBMP-2 was adsorbed on the HAP and Mg-HAP surfaces as discussed in the adsorption stability study. C2C12 cells were cultured on the prepared surfaces at a density of 1 × 10^5^ cells per well (24-well) for 6 h. After extracted by RIPA lysis buffer, samples were subjected to 10% SDS–PAGE and transferred to PVDF membranes. The membranes were blocked by 5% BSA at room temperature for 60 min and incubated with primary antibodies (anti-Smad1/5/8, anti-phospho-Smad1/5/8, and anti-GAPDH) at 4 °C overnight. Then the membranes were incubated with secondary antibodies (HRP-conjugated anti-rabbit IgG) for 2 h. Following, the membranes were visualized and analyzed using the ECL plus reagents by Image Quant LAS 4000 (GE, USA).

#### Quantitative real time PCR study

Expression of rhBMP-2-mediated genes and BMPRs genes was further quantitatively analyzed using the real time PCR assay. C2C12 cells were cultured on respective surfaces at a density of 1 × 10^5^ cells per well (24-well) for 12 h. Total RNA was extracted using Trizol reagent following manufacturer’s instructions. First-stranded cDNA was synthesized with PrimeScript RT reagent Kit. After diluting cDNA by ten-fold in sterile distilled water, 4 μL aliquot of the diluted cDNA was subjected to real-time PCR using SYBR Premix Ex Taq. Subsequently, real-time PCR was performed by Bio-Rad real-time PCR system (Bio-Rad, Hercules, USA) according to manufacturer’s instructions. PCR primer pairs were designed based on the sequences of different exons of the corresponding genes (Table S4, Supporting Information). The conditions of real-time PCR were as following: 95 °C for 30 s followed by 40 cycles at 95 °C for 5 s and 60 °C for 34 s. ΔCT was used to calculate the differences between target and control CT values for each sample: ΔCT = CT (target)−CT (control). The comparative expression level (fold change) was obtained by transforming the logarithmic values into absolute values using 2^−ΔCT^.

#### Cell attachment

The cellular adhesion parameters (cellular morphology, focal adhesion, cell-material contact area, and cellular perimeter) of HAP/rhBMP-2 and Mg-HAP/rhBMP-2 were measured by a CLSM. Briefly, rhBMP-2 was adsorbed on the HAP and Mg-HAP surfaces as discussed in the adsorption stability study. The C2C12 cells (1 × 10^4^ cells/well, 24-well plate) were seeded on the prepared surfaces. In addition, C2C12 cells directly cultured on the HAP and Mg-HAP surfaces were used as controls. After 12 h incubation, the surfaces were removed from the culture plates, rinsed shortly in 37 °C PBS and fixated with 10% neutral PBS buffered formalin. After fixation, the surfaces were washed twice with warm PBS, permeabilized with 0.1% Triton X-100, and washed twice again with PBS. Following this, the cells were incubated for 30 min at room temperature with a 1% BSA blocking agent and washed twice with PBS buffer. 400 μL of mouse monoclonal anti-vinculin (5 μg/mL) was added to the cells and incubated overnight at 4 °C and then washed three times with PBS. 200 μL of TRITC-conjugated goat anti-mouse IgG (10 μg/mL) and 200 μL of FITC-conjugated phalloidin (2 μg/mL) were added to the surfaces and incubated for 60 min at room temperature. Cells were thereafter washed three times with PBS and finally incubated for 10 min at room temperature with 200 μL of 0.5 μM DAPI and washed three times with PBS. The stained cells were kept in the dark at 4 °C until the substrates were investigated by CLSM. 50–60 images at 600X magnification were acquired at random on each surface.

### Molecular dynamics simulation

#### Computational setup

The atomistic MD and SMD simulations were conducted with GROMACS 5.0.4 applying the OPLS-AA force field. All calculations were carried out under periodic boundary conditions in explicit TIP3P water with a cut-off distance of 12 Å for van der Waals and a value of 12 Å for the separation of the direct and reciprocal space summations. Long-range electrostatics was computed with the particle mesh Ewald summation.

#### Surface model

Molecular models for HAP and Mg-HAP were developed by duplication of small unit cells using Materials Studio 7.0. The initial atomic coordinates of HAP (P6_3_/m, unit cell parameters *a* = *b* = 0.943 nm and *c* = 0.688 nm) was extracted from the American Mineralogist Crystal Structure Database. The (0 0 1) face of HAP is our surface of interest as this face is the major surface of HAP materials and has been extensively investigated in numerous modeling studies^36^,^37^,^39^. Given that natural HAP nanoparticles have lower crystallinity than a HAP cell (HAPC), the HAPC underwent 1 ns of MD simulation and was then utilized to develop the super-cell. For the HAP model, a super-cell (15 × 15 × 4 units) of HAPC was built. For the Mg-HAP model, a uniform substitution of Ca^2+^ into Mg^2+^ (2.2 at% of total Ca^2+^, same content as the experimental Mg-HAP surface) at Ca(II) sites was performed on the HAP model, given that Mg^2+^ preferentially replaces at Ca(II) sites of HAPC^30^. The HAP and Mg-HAP models were fixed in all simulations.

#### Protein

The initial configuration of the protein was obtained from Protein Database Bank (PDB, ID code = 3BMP). In this monomer structure, coordinates of the first 8 residues corresponding to the N-terminus are missing^41^. A homo-dimer (natural bioactivity form) linked with inter-chain disulfide bridges between the Cys 78 residues of each monomer was developed and utilized in all simulations. The resulting structure had a total charge of -4 e, which was neutralized by adding ions in the protein-surface system setup part. Given that the coordinates of rhBMP-2 are currently unavailable in PDB and the BMP-2 structure of 3BMP has been extensively utilized in modelling simulations^36^,^39^,^40^, BMP-2 (homo-dimer) was applied in the simulation part instead of rhBMP-2.

#### Protein-surface system setup

Proteins can interact with material surfaces at different orientations, which could lead to distinct dynamic adsorption behaviors. As the module of BMP-2 is roughly considered as a rectangular box, six initial scenarios with each face of the protein box lying on the material surface in the z-axis were used (as shown in Fig. 7, inserts). In this study, the two end-on and four side-on orientations are defined as End1, End2, Side1, Side2, Side3, and Side4. The initial separation distances between BMP-2 (centre of mass) and the surface were set as approximate 10 nm and 8 nm for the end-on and side-on orientations, respectively. Considering the release of Ca^2+^ and Mg^2+^ measured by ICP-OES and the box size of system (24.5 × 24.5 × 18 nm), ions were added to neutralize the systems. For the HAP systems, 4 Ca^2+^ and 4 Cl^−^ were randomly added and for the Mg-HAP systems, 4 Ca^2+^, 2 Mg^2+^, and 8 Cl^−^ were randomly added. Energy minimization was performed by using the steepest descent method (stop at force <1000 kJ/mol/nm). To further equilibrating the system, a 100 ps of NVT ensemble (T = 300 K) was conducted, followed with NPT ensemble (P = 1 bar) for another 100 ps.

#### MD and SMD simulations

A hybrid approach combining MD and SMD simulations was performed to explore the mechanism of adsorption of BMP-2 on the HAP and Mg-HAP surfaces. In 750 ps of SMD simulation, artificial forces were applied to the protein backbone, pushing it towards the surface with constant velocity (0.01 nm/ps) and with a spring constant of 1000 kJ/(mol nm^2^). The binding energy was recorded with respect to the distance between BMP-2 and the surface. The configuration of system at the adsorption state, which had the minimum binding energy, was selected and further relaxed with 2 ns of MD simulation. The Rg and RMSD of BMP-2 were quantified throughout the SMD and MD simulations. In addition, the RMSF of each residue of BMP-2 was also investigated over the whole process.

#### Stability of cysteine-knots

Each BMP-2 monomer has 7 cysteines, 6 of them constitute 3 intramolecular disulfides^41^, which are known as cysteine-knots (Cys43/Cys111, Cys47/Cys113, and Cys14/Cys79). One of them (Cys78) forms an intermolecular disulfide with the other monomer, constituting a dimer. The Cysteine-knot is crucial for the stability of conformation and thus for the bioactivity of BMP-2^40^,^41^,^44^. To establish the connection between bioactivity of BMP-2 and molecular conformation, we measured the RMSF of cysteine-knots of BMP-2 adsorbed on the HAP and Mg-HAP surfaces. The RMSF of cysteine-knots was defined as follows:


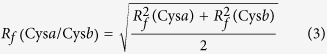


where R_*f*_ (Cys*a*/Cys*b*) stands for the RMSF of cysteine-knot of (Cys*a*/Cys*b*), and R_*f*_ (Cys*a*) and R_*f*_ (Cys*b*) stands for the RMSF of residue of Cys*a* and Cys*b*, respectively. Cys*a* and Cys*b* stands for the residue *a* and *b* of BMP-2, respectively.

### Statistical analysis

All numerical data was presented as the mean ± standard deviation (SD), with similar results obtained in each experiment. Significant differences were analyzed with independent sample Student t-tests. Values of p < 0.05 were accepted as statistically significant.

## Additional Information

**How to cite this article**: Huang, B. *et al*. Facilitated receptor-recognition and enhanced bioactivity of bone morphogenetic protein-2 on magnesium-substituted hydroxyapatite surface. *Sci. Rep*. **6**, 24323; doi: 10.1038/srep24323 (2016).

## Supplementary Material

Supplementary Information

## Figures and Tables

**Table 1:** Thickness and Ca/P ratio of coatings, RMS (root-mean-square roughness), surface charge, and water contact angle of the HAP and Mg-HAP surfaces (n = 5).

	HAP	Mg-HAP
Thickness (nm)	28.2 ± 2.6	30.4 ± 3.5
Ca/P ratio	1.71 ± 0.05	1.64 ± 0.06
RMS (nm)	5.2 ± 0.7	6.1 ± 0.9
Surface charge (mV)	−1.8 ± 0.3	−1.6 ± 0.2
Water contact angle (°)	74.7 ± 2.4	72.2 ± 1.3

**Table 2:** Surface mass density and visco-elastic property of adsorbed rhBMP-2 and BMPRs-recruitment to rhBMP-2 layer on the HAP and Mg-HAP surfaces (n = 5).

	HAP	Mg-HAP
Δm_rhBMP-2_(ng/cm^2^)	63.7 ± 2.5	55.2 ± 1.8
(ΔD/Δf)_rhBMP-2_(10^−8^Hz^−1^)	3.94 ± 0.22	6.73 ± 0.32
Δm_BMPR-IA_(ng/cm^2^)	30.1 ± 1.1	56.6 ± 2.1
Δm_BMPR-IB_(ng/cm^2^)	7.8 ± 0.7	14.2 ± 1.4
Δm_BMPR-II_(ng/cm^2^)	19.8 ± 1.4	18.8 ± 1.1
Δm_ActR-I_(ng/cm^2^)	5.7 ± 1.1	5.3 ± 0.9
Δm_ActR-II_(ng/cm^2^)	4.7 ± 0.6	4.9 ± 0.8
Δm_ActR-IIB_(ng/cm^2^)	6.4 ± 1.3	5.6 ± 1.2

**Table 3:** Rg shifts (nm) of BMP-2 molecule (in 6 orientations) adsorbed on the HAP and Mg-HAP surfaces compared to the initial configuration (n = 5).

Orientation	HAP	Mg-HAP
End1	−0.193 ± 0.005	−0.246 ± 0.004
End2	−0.142 ± 0.005	−0.288 ± 0.011
Side1	0.149 ± 0.006	0.056 ± 0.008
Side2	0.091 ± 0.003	0.238 ± 0.012
Side3	0.138 ± 0.004	0.060 ± 0.006
Side4	0.115 ± 0.009	0.159 ± 0.006
