# Supplementary material for: Facilitated receptor-recognition and enhanced bioactivity of bone morphogenetic protein-2 on magnesium-substituted hydroxyapatite surface
Source: Sci Rep. 2016 Apr 14;6:24323. doi: 10.1038/srep24323 (PMC4830968; doi:10.1038/srep24323)
Supplement: Supplementary Information [file srep24323-s1.pdf]

Supporting Information

Facilitated receptor-recognition and enhanced bioactivity of bone morphogenetic protein-2 on magnesium-substituted hydroxyapatite surface

Baolin Huang [a, b, d], Yuan Yuan [a, b, c, ***], Tong Li [d], Sai Ding [b, c], Wenjing Zhang [a, c], Yuantong Gu [d, **], Changsheng Liu [a, b, c, *]

[a] *State Key Laboratory of Bioreactor Engineering, East China University of Science and Technology, Shanghai 200237, PR China*

[b] *Key Laboratory for Ultrafine Materials of Ministry of Education, East China University of Science and Technology, Shanghai 200237, PR China*

[c] *Engineering Research Center for Biomedical Materials of Ministry of Education, East China University of Science and Technology, Shanghai 200237, PR China*

[d] *School of Chemistry, Physics and Mechanical Engineering, Queensland University of Technology, 2 George St, Brisbane, QLD 4001, Australia*

Table S1 Minimal binding energy (kJ/mol) of BMP-2 adsorbed on the HAP and Mg-HAP models in 6 orientations during SMD process (n = 5)

Orientation	HAP	Mg-HAP
End1	-598 ±37	-559 ±69
End2	-288 ±32	-569 ±92
Side1	-1807 ±58	-1396 ±58
Side2	-1011 ±70	-531 ±54
Side3	-254 ±31	-789 ±55
Side4	-287 ±30	-927 ±36

Table S2 Separation distance (nm) between BMP-2 (centre of mass, in 6 orientations) and surface at the state of minimal binding energy on the HAP and Mg-HAP models during SMD process (n = 5)

Orientation	HAP	Mg-HAP
End1	4.48 ±0.08	4.85 ±0.07
End2	4.88 ±0.09	4.70 ±0.07
Side1	3.22 ±0.06	3.38 ±0.04
Side2	3.14 ±0.05	3.31 ±0.06
Side3	3.59 ±0.07	3.30 ±0.04
Side4	3.61 ±0.08	3.35 ±0.05

As indicated in Table S1 and Table S2, among all the orientations, the Side1 orientation was the most preferential orientation for adsorption of BMP-2. Basically, the separation distances between BMP-2 and the surface for side-on orientations were notably shorter ($p < 0.05$) than those for end-on orientations. In the Side1 and Side2 orientations, the separation distances increased in a trend of HAP < Mg-HAP. In the Side3 and Side4 orientations, however, the separation distances exhibited the opposite trend. The separation distances for end-on orientations among the HAP and Mg-HAP surfaces were similar, except a statistically shorter separation distance was reached for the End1 orientation of BMP-2 on the HAP surface.

Table S3 Residues of BMP-2 with a high value of RMSF (> 0.25 nm) during the adsorption on the HAP and Mg-HAP surfaces

Orientation	HAP	Mg-HAP
End1	Arg9, Leu10, Ser24-Val26, Pro35, Pro36, Asp53, Asp93-Val99, Arg9’, Phe49’, Asp53’-Asn56’, Lys73’	Arg9, Leu10, Ser24-Val26, Trp28, Asp30-Pro36, Ile87-Leu90, Glu96-Val98, Arg9’-Lys11’, Phe49’, Leu51’, Ala52’, His54’-Asn56’
End2	Arg9, Lys11, Glu94-Glu96, Glu94’-Glu96’	Asp53-Leu55, Thr58, Asn59, Glu94, Arg9’, Leu10’, Tyr38’
Side1	Glu94-Glu96, Val98, Val99, Arg114, Trp31’, Tyr38’, His60’	His39, Asp53, Glu94, Asn95, Glu109, Leu51’, Asn59’, His60’, Glu94’
Side2	Arg9, Lys11, Phe23-Val33, Pro36, Asn68, Lys73, Ile74, Glu94-Lys97, Arg9’, Leu10’, Asp22’-Trp28’, Trp31’, Asn68’-Lys76’	Arg9, Leu10, Tyr20-Tyr38, Val63, Thr65-Val67, Ser69, Val70, Ser72, Lys73, Leu84, Leu92, Glu94-Val98, Lys101, Met106-Val108, Arg9’-Ser13’, Tyr20’, Asp22’-Val26’, His44’, Pro48’, Phe49’, His60’, Ile62’-Ile74’, Glu96’
Side3	Asp53-Asn56, Arg9’, Ser69’, Val70’, Glu94’-Lys97’	Tyr20, Phe49-Leu51, His54-Asn56, Gln64, Lys73, Tyr91, Arg9’, Glu94’-Leu100’
Side4	Arg9, Trp28, Lys73, Glu94-Glu96, Asp53’, His54’	Trp28, Trp31’, Phe49’, Ala52’-Asn59’, Ala61’, Leu66’, Val67’, Arg114’

Table S4 Parameters of primer used for Quantitative real time PCR assay

Gene	Direction	Sequence (5’ - 3’)
Id1	Forward	CATGAACGGCTGCTACTCAC
	Reverse	GAACACATGCCGCCTCG
Runx2	Forward	CGGCCCTCCCTGAACTCT
	Reverse	TGCCTGCCTGGGATCTGT
OCN	Forward	CTGACAAAGCCTTCATGTCCAA
	Reverse	GCGGGCGAGTCTGTTCACTA
BMPR-IA	Forward	TCGTCGTTGTATTACAGGAG
	Reverse	TTACATCCTGGGATTCAACC
BMPR-IB	Forward	GCTTTGGACTCATCCTCTGG
	Reverse	CACTGGGCAGTAGGCTAACG
BMPR-II	Forward	GGTAGATAGGAGGGAACGGC
	Reverse	CACTGCCATTGTTGTTGACC
ActR-I	Forward	AGATGACGTGTAAGACCCCG
	Reverse	ATACTTCTCCATAGCGGCCC
ActR-II	Forward	GTGCAGAGAAAAGAGGCACC
	Reverse	TATCCTCAGAAATGCGTCCC
ActR-IIB	Forward	GAACATCATCACGTGGAACG
	Reverse	ATGTACTCATCGACAGGCCC
GAPDH	Forward	GTCGTGGAGTCTACTGGTGTC
	Reverse	GAGCCCTTCCACAATGCCAAA

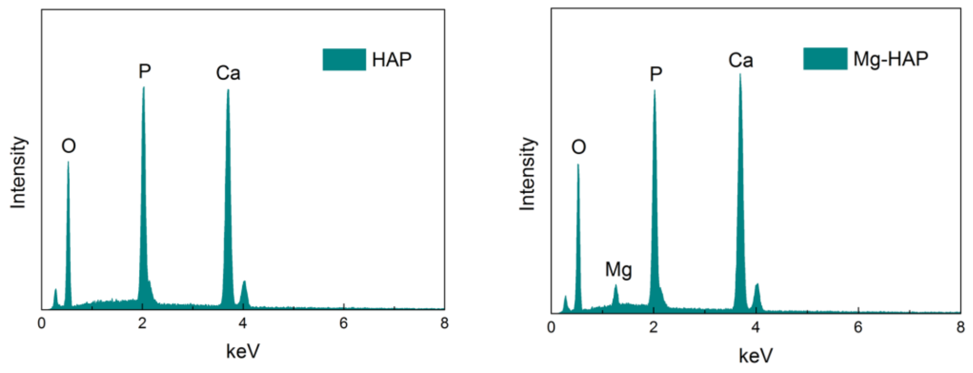

Figure S1 EDS patterns of the HAP and Mg-HAP nanoparticles.

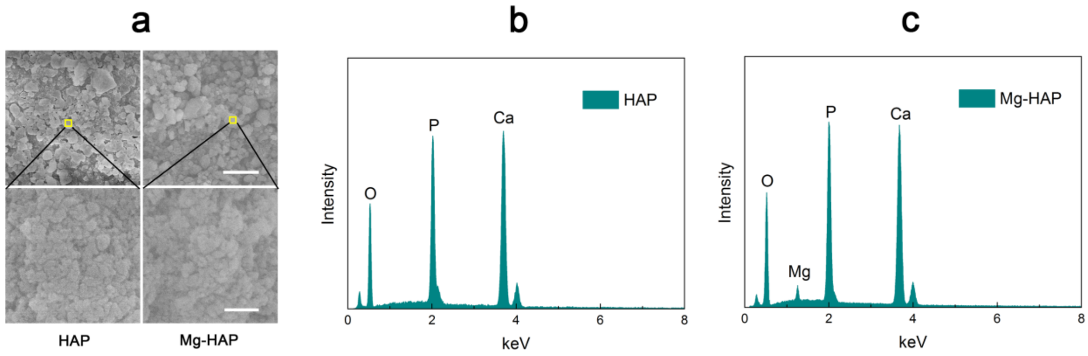

Figure S2 (a) SEM images of the HAP and Mg-HAP surfaces. Scale bar is 10 um for the up-row and 200 nm for the down-row. (b-c) EDS patterns of the HAP and Mg-HAP coatings.

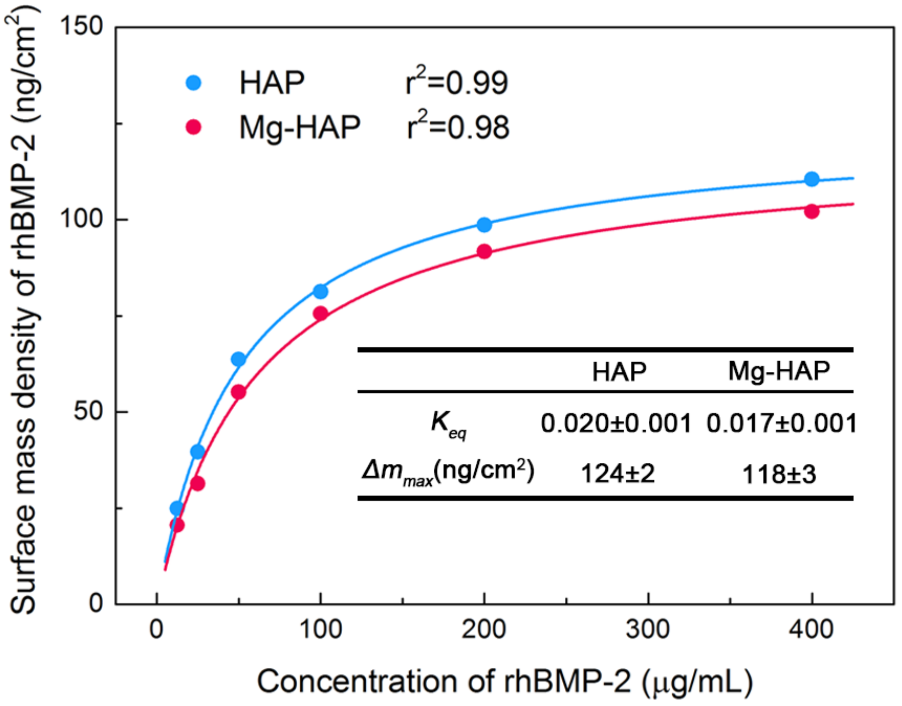

Figure S3 Isothermal adsorption of rhBMP-2 at various concentrations on the HAP and Mg-HAP surfaces. The curves are fitted with the Langmuir adsorption equation. The relative adsorption parameters are presented in the insert table. The equilibrium binding constant and maximum binding capability of rhBMP-2 on the Mg-HAP surface is slightly lower than the HAP surface.

The Langmuir adsorption equation[1,2] is given as following:

$$\Delta \mathrm{m}_{\mathrm{rhBMP-2}} = \frac{\Delta \mathrm{m}_{\mathrm{max}} K_{eq}\, c}{1+K_{eq}\, c} \qquad (1)$$

where K_{eq} is the equilibrium binding constant, c is the concentration of rhBMP-2, $\Delta m_{rhBMP-2}$ (ng/cm^2) is the surface mass density of rhBMP-2 at a given concentration of c, and Δm_{max} (ng/cm^2) is the mass density of rhBMP-2 at its full monolayer coverage.

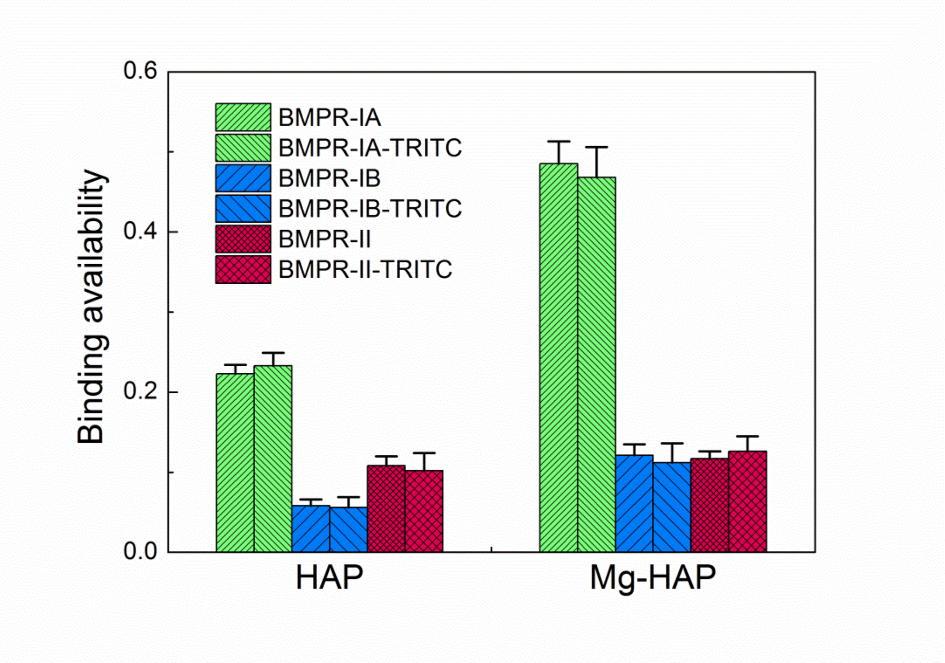

Figure S4 Comparison of BMPRs-TRITC/rhBMP-2-FITC binding availability and BMPRs/rhBMP-2 binding availability on the HAP and Mg-HAP surfaces. Values are shown as mean ± standard error of the mean from 5 data points (n = 5).

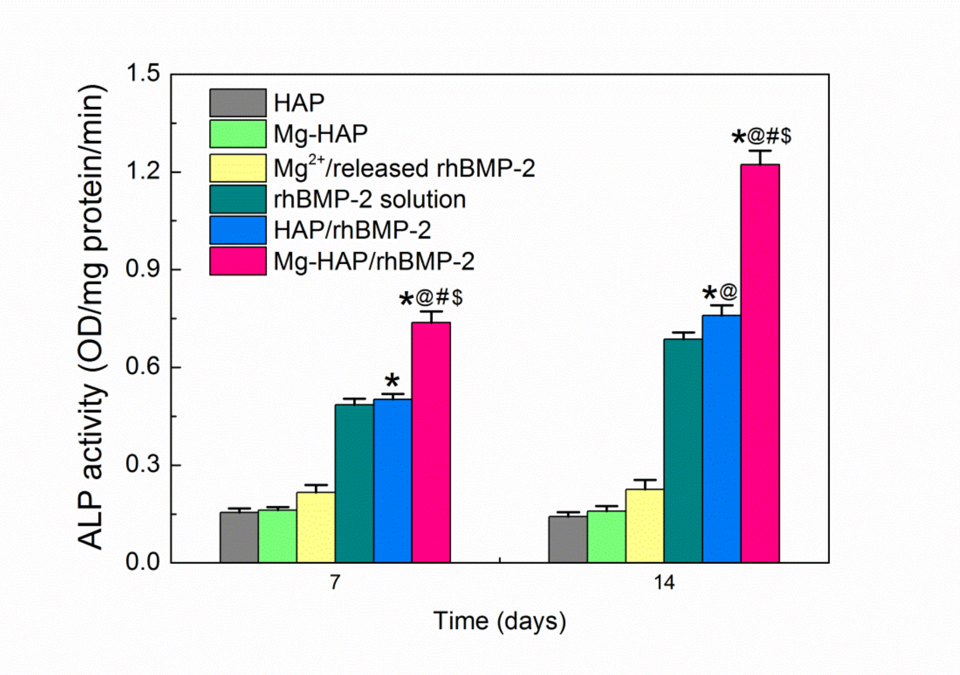

Figure S5 ALP activity assay of BMSCs. The HAP and Mg-HAP surfaces without rhBMP-2 were acted as negative controls. The rhBMP-2 solution representing culture medium containing fresh rhBMP-2 at the similar content to that calculated from the QCM-D study was used as a positive control. The Mg^{2+}/released rhBMP-2 represents culture medium contain Mg^{2+} ions (released amount) and fresh rhBMP-2 (released amount). Values are shown as mean ± standard error of the mean from 5 data points (n = 5). * $p < 0.05$, compared with the corresponding surfaces without rhBMP-2; # $p < 0.05$, compared with HAP/rhBMP-2; @ $p < 0.05$, compared with rhBMP-2 solution; $ $p < 0.05$ compared with Mg^{2+}/released rhBMP-2.

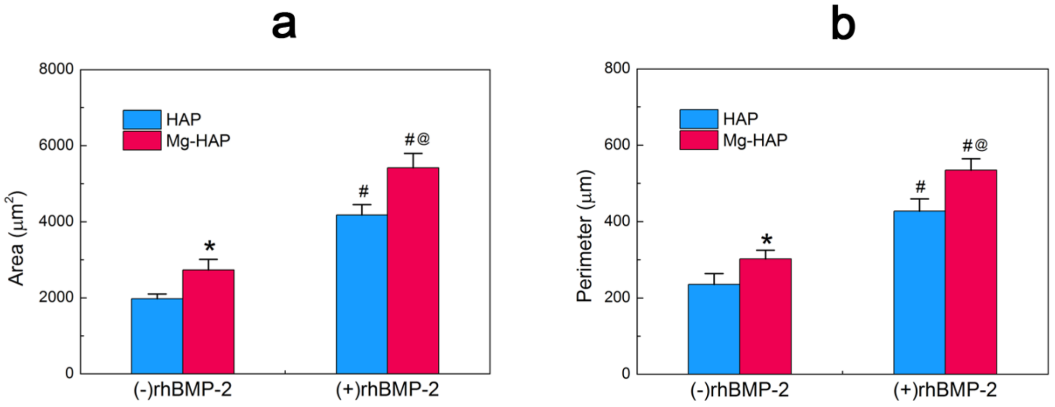

Figure S6 Cell-surface contact area (a) and cellular perimeter (b) of C2C12 cells cultured on the rhBMP-2-adsorbed/unadsorbed HAP and Mg-HAP surfaces. Values are shown as mean ± standard error of the mean from 50 data points (n = 50). $^{*}p < 0.05$, compared with the HAP surface without rhBMP-2. $^{@}p < 0.05$, compared with the rhBMP-2-adsorbed HAP surface. $^{\#}p < 0.05$, compared with the corresponding surfaces without rhBMP-2.

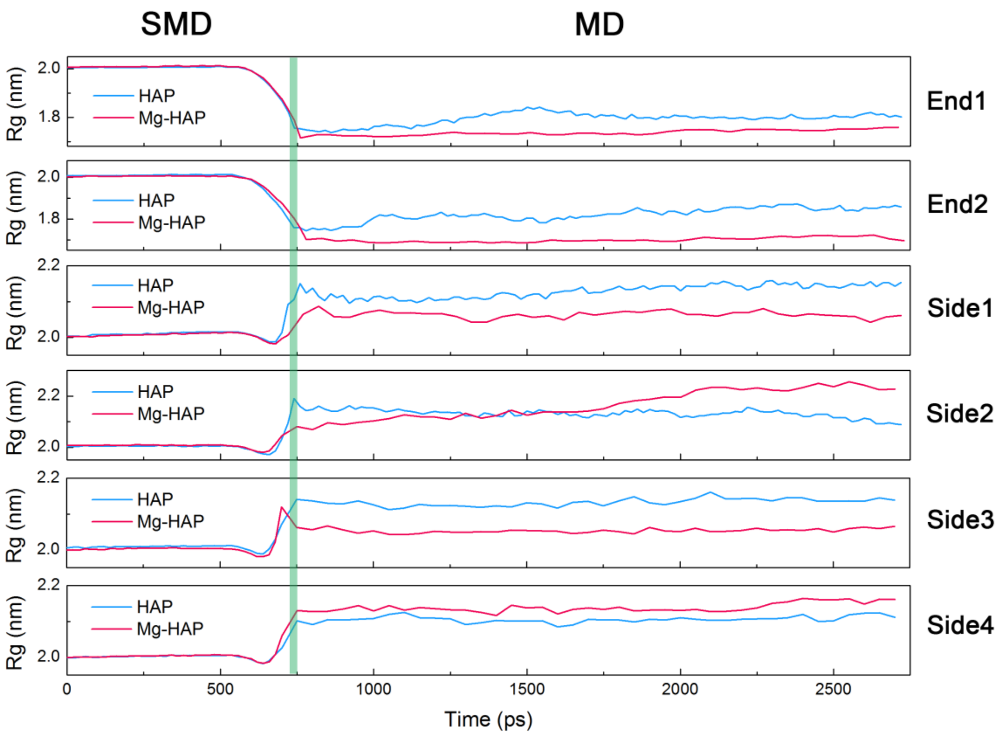

Figure S7 Rg of BMP-2 against simulation time for different orientations. SMD simulation: 0 to 750 ps; MD simulation: 750 to 2750 ps.

As shown in Fig. S7, it is found that the Rg of BMP-2 stabilized at 2.0 nm from 0 to 600 ps in the SMD procedure. A remarkable shift of Rg of BMP-2 is accomplished from 600 to 750 ps in the SMD simulation. Further changes of Rg of BMP-2 are also achieved in the full MD procedure (750 to 2750 ps).

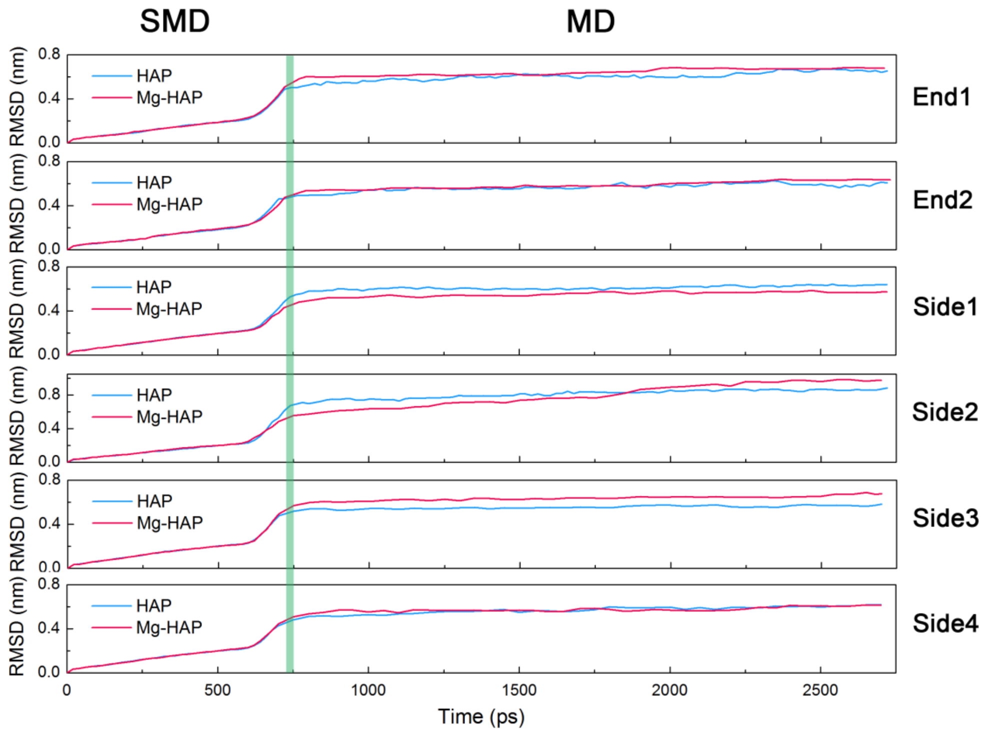

Figure S8 RMSD of backbone atoms of BMP-2 (in 6 orientations) adsorbed on the HAP and Mg-HAP surfaces for the MD and SMD simulations.

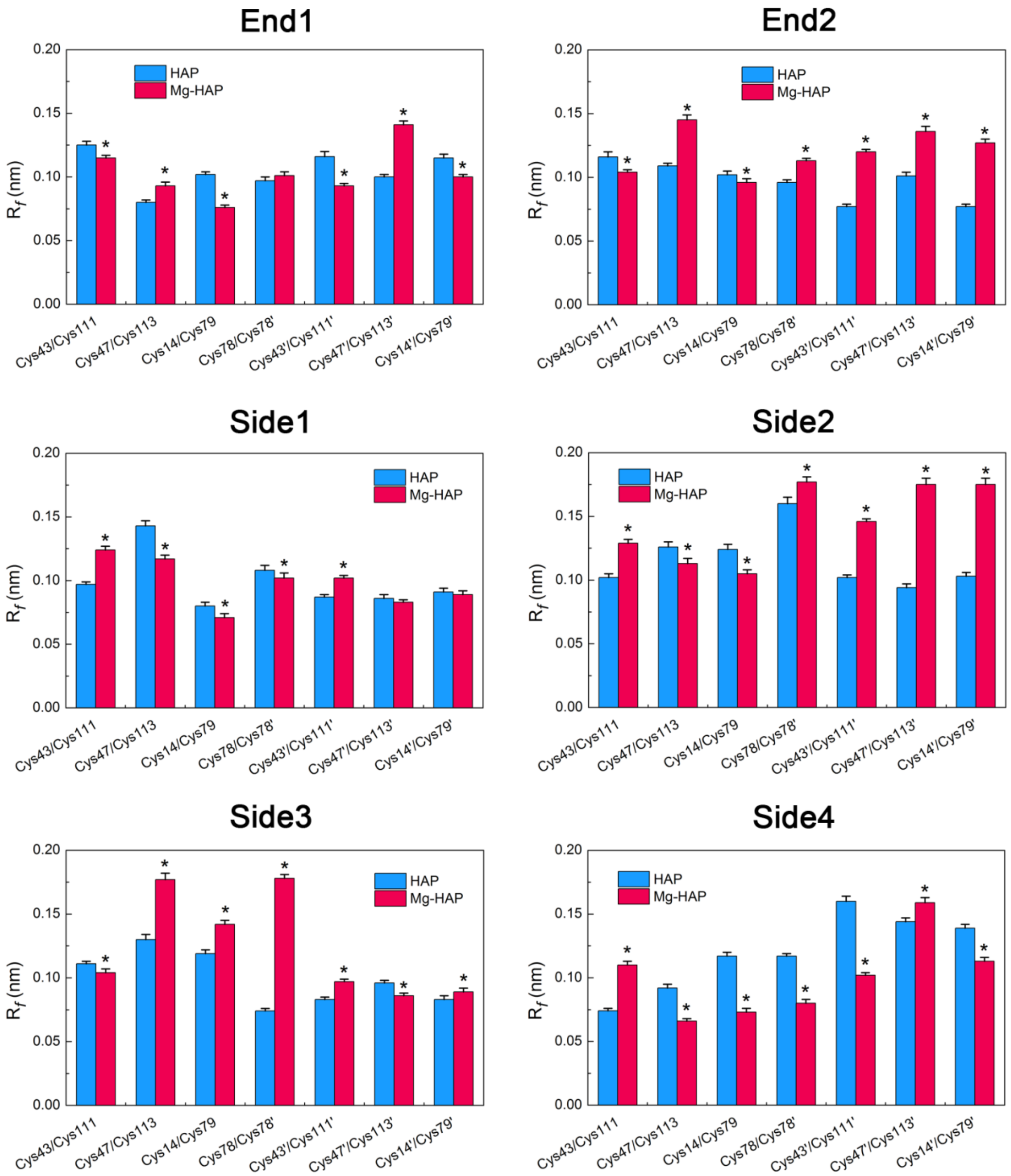

Figure S9 RMSF (R_f) of cysteine-knots of BMP-2 adsorbed on the HAP and Mg-HAP surfaces with various orientation. Values are shown as mean ± standard error of the mean from 5 data points (n = 5). * $p < 0.05$, compared with the HAP surface.

As shown in Fig. S9, the values of RMSF of cysteine-knots of BMP-2 are relative low. In the End1 orientation, 4 cysteine-knots (Cys43/Cys111, Cys14/Cys79, Cys43'/Cys111', and Cys14'/Cys79') showed significantly lower ($p < 0.05$) RMSF on the Mg-HAP surface than that on the HAP surface. The intermolecular cysteine-knot (Cys78/Cys78') exhibited a similar value of RMSF on the HAP and Mg-HAP surfaces. In the End2 orientation, only 2 cysteine-knots (Cys43/Cys111 and Cys14/Cys79) exhibited significantly lower ($p < 0.05$) RMSF on the Mg-HAP surface than that on the HAP surface. In the Side1 orientation, 3 cysteine-knots (Cys47/Cys113, Cys14/Cys79, and Cys78/Cys78') showed significantly lower ($p < 0.05$) RMSF on the Mg-HAP surface than that on the HAP surface. There was no significantly difference ($p > 0.05$) for the value of RMSF of 2 cysteine-knots (Cys47'/Cys113' and Cys14'/Cys79') on the HAP and Mg-HAP surfaces. In the Side2 orientation, only 2 cysteine-knots (Cys47/Cys113 and Cys14/Cys79) showed significantly lower ($p < 0.05$) RMSF on the Mg-HAP surface than that on the HAP surface. In the Side3 orientation, only 2 cysteine-knots (Cys43/Cys111 and Cys47'/Cys113') exhibited lower ($p < 0.05$) RMSF on the Mg-HAP surface than that on the HAP surface. In the Side4 orientation, 5 cysteine-knots (Cys47/Cys113, Cys14/Cys79, Cys78/Cys78', Cys43'/Cys111', and Cys14'/Cys79') exhibited remarkably lower ($p < 0.05$) RMSF on the Mg-HAP surface than that on the HAP surface. Together, these results indicated that the cysteine-knots of BMP-2 on the Mg-HAP surface are more stable than that on the HAP surface.

References

1 Boix, T. *et al.* Adsorption of recombinant human bone morphogenetic protein rhBMP-2m onto hydroxyapatite. *J Inorg Biochem* **99**, 1043-1050, doi:10.1016/j.jinorgbio.2005.01.011 (2005).

2 Dolatshahi-Pirouz, A., Jensen, T., Foss, M., Chevallier, J. & Besenbacher, F. Enhanced Surface Activation of Fibronectin upon Adsorption on Hydroxyapatite. *Langmuir* **25**, 2971-2978 (2009).
